# The E3 ligase TRIM56 is a host restriction factor of Zika virus and depends on its RNA-binding activity but not miRNA regulation, for antiviral function

**DOI:** 10.1371/journal.pntd.0007537

**Published:** 2019-06-28

**Authors:** Darong Yang, Nan L. Li, Dahai Wei, Baoming Liu, Fang Guo, Husni Elbahesh, Yunzhi Zhang, Zhi Zhou, Guo-Yun Chen, Kui Li

**Affiliations:** 1 Department of Microbiology, Immunology and Biochemistry, University of Tennessee Health Science Center, Memphis, TN, United States of America; 2 Children’s Foundation Research Institute at Le Bonheur Children’s Hospital, Department of Pediatrics, University of Tennessee Health Science Center, Memphis, TN, United States of America; 3 Baruch S. Blumberg Institute, Doylestown, PA, United States of America; 4 Department of Infectious Diseases, the Second Affiliated Hospital of Chongqing Medical University, Chongqing, China; Oxford University Clinical Research Unit, VIET NAM

## Abstract

Infection by Zika virus (ZIKV) is linked to microcephaly and other neurological disorders, posing a significant health threat. Innate immunity is the first line of defense against invading pathogens, but relatively little is understood regarding host intrinsic mechanisms that guard against ZIKV. Here, we show that host tripartite motif-containing protein 56 (TRIM56) poses a barrier to ZIKV infection in cells of neural, epithelial and fibroblast origins. Overexpression of TRIM56, but not an E3 ligase-dead mutant or one lacking a short C-terminal portion, inhibited ZIKV RNA replication. Conversely, depletion of TRIM56 increased viral RNA levels. Although the C-terminal region of TRIM56 bears sequence homology to NHL repeat of TRIM-NHL proteins that regulate miRNA activity, knockout of Dicer, which abolishes production of miRNAs, had no demonstrable effect on ZIKV restriction imposed by TRIM56. Rather, we found that TRIM56 is an RNA-binding protein that associates with ZIKV RNA in infected cells. Moreover, a recombinant TRIM56 fragment comprising the C-terminal 392 residues captured ZIKV RNA in cell-free reactions, indicative of direct interaction. Remarkably, deletion of a short C-terminal tail portion abrogated the TRIM56-ZIKV RNA interaction, concomitant with a loss in antiviral activity. Altogether, our study reveals TRIM56 is an RNA binding protein that acts as a ZIKV restriction factor and provides new insights into the antiviral mechanism by which this E3 ligase tackles flavivirus infections.

## Author summary

The E3 ligase TRIM56 was previously shown to inhibit the replication of several viruses in the family Flaviviridae, including dengue virus serotype 2, yellow fever virus and bovine viral diarrhea virus, but had not demonstrable antiviral effect against hepatitis C virus, a hepatotropic virus in the same family. Nonetheless, the antiviral mechanism remains unclear and whether TRIM56 restricts other flaviviruses remains to be determined. In this study we demonstrated that TRIM56 inhibits ZIKVs of Asian and African lineages and a dengue virus serotype 1 replicon. We additionally uncovered that TRIM56 is an RNA-binding protein and that a portion of the C-terminal NHL-like domain mediates the association of TRIM56 with ZIKV RNAs in infected cells. Importantly, the RNA-binding activity of TRIM56 was found to be required for its antiviral function, although it alone is insufficient. In contrast, TRIM56 restricted ZIKV in Dicer-deficient cells, indicating an antiviral mechanism independent of miRNA regulation, a function known to be associated with NHL-containing proteins. In aggregate, our work identifies TRIM56 as a novel restriction factor of ZIKV and sheds new lights on the antiviral mechanism of TRIM56 against flaviviruses.

## Introduction

Zika virus (ZIKV) is a small, enveloped RNA virus classified within the family Flaviviridae, genus flavivirus, which also includes medically important pathogens such as dengue virus (DENV), West Nile virus (WNV), Japanese encephalitis virus (JEV), and yellow fever virus (YFV) [1], among others. As with other flaviviruses, ZIKV possesses a ~11-kb long, single-stranded RNA genome of positive polarity. After infecting susceptible cells, the viral genomic RNA is released into the cytoplasm and subsequently translated to a large polyprotein that is cleaved into three structural proteins (C, prM/M, and E) and seven non-structural (NS) proteins (NS1, NS2A, NS2B, NS3, NS4A, NS4B, and NS5) by a combination of viral and host proteases [2]. While the structural proteins make up the virions, viral NS proteins along with the genomic RNA template assemble into replicase complexes on cytoplasmic membrane structures, synthesizing nascent viral RNAs that are packaged into progeny viruses and released from infected cells to go on infecting naïve cells [2]. The replication and pathogenesis of ZIKV depend upon its intricate interplay with host factors, much of which, however, remains to be elucidated.

Based on the phylogeny of viral sequences, ZIKV is classified into two major lineages, African and Asian [3]. First isolated in 1947 in Uganda [4], ZIKVs of African lineage have rarely been associated with human cases, which typically exhibit an acute febrile illness. However, the outbreaks in the past decade of viruses of Asian lineage, not only greatly outnumbered the human infections known to be caused by the African virus but are notorious for their serious complications. Mounting evidence suggests that infection by ZIKV of Asian lineage is linked to congenital defects including microcephaly and spontaneous abortion [5–7] and to Guillain-Barre syndrome and thrombocytopenia in adults [8, 9]. Precisely how ZIKV causes these congenital and neurological disorders is unclear, but both viral and host factors have been suggested to play a role [10]. Of note, ZIKV replicates in neural progenitor cells, causing cell cycle arrest and/or death of neurons [10, 11]. Although mosquito bite is the most common route of ZIKV infection, ZIKV can also spread from person to person via sexual contact or vertically from pregnant woman to fetus [12–15]. Consistent with these epidemiological findings, previous studies have revealed that ZIKV infects human skin cells, placental cells, endometrial stromal cells, *etc…* [11, 16–18]. Further studies on tissue tropism of the virus and factors regulating viral replication in and host responses of susceptible cell types are warranted, as they will yield novel insights into mechanisms of ZIKV pathogenesis and may identify therapeutic targets.

Host cells are equipped with exquisite, intrinsic mechanisms to fend off invading viruses, which could be harnessed for developing antivirals against flaviviruses, including ZIKV. The mammalian innate immune system constitutes the first line of defense against invading microorganisms including viral pathogens. A hallmark of the intrinsic antiviral responses is the rapid induction of interferons (IFNs). Once produced, IFNs act in a paracrine and/or autocrine fashion, signaling through cell surface IFN receptors and downstream Jak-Stat pathway, culminating in the upregulation of hundreds of IFN-stimulated gene (ISG) products that collectively establish an antiviral state, reining in viral replication and spread [19]. Flaviviruses are known to subject to innate immune control and ZIKV is no exception [20]. It has been recently reported that mice lacking *Ifnar1* or triply deficient for *Irf3/Irf5/Irf7* supported heightened ZIKV replication, concomitant with developing neurological diseases [21]. Of the ~ 300 known ISGs, IFITM1, IFITM3 and Viperin have been shown to inhibit ZIKV [22, 23]. However, much remains to be learned regarding ZIKV restriction factors—inhibitory host factors against ZIKV, particularly those that are constitutively expressed and whose expression is not or only moderately induced by IFNs.

We have previously demonstrated that TRIM56, a RING-type E3 ligase of the tripartite motif protein family, puts a check on intracellular RNA replication of DENV serotype 2 (DENV2), YFV, and bovine viral diarrhea virus (BVDV) in cell culture [24, 25]. Although the inhibitory effects on these viruses invariably rely upon the E3 ligase activity as well as the C-terminal integrity of TRIM56 [24, 25], the underlying mechanism remains elusive. In addition, whether the antiviral spectrum of TRIM56 can be extended to other viruses within the flavivirus genus, especially ZIKV, is an intriguing and important question to answer.

In this study, we demonstrate that TRIM56 exerts a direct antiviral effect on ZIKV infection in human cells of fibroblast-, epithelial-, and neural-origins and that both the E3 ligase activity and C-terminal portion of TRIM56 are critical for restricting ZIKV. Although the C-terminal region of TRIM56 bears sequence homology with the NHL repeat motif of several other TRIM proteins [26] that bind miRNAs and/or mRNAs [27–29], we found that the inhibition on ZIKV replication by TRIM56 was not undercut in Dicer-deficient cells, indicative of a miRNA-independent antiviral mechanism. Interestingly, we revealed that TRIM56 was associated with ZIKV RNA in infected cells via its C-terminal portion and such capability was required for its antiviral function. Altogether, our work illustrates that TRIM56 is an intrinsic host restriction factor of ZIKV and shed new lights on the mechanism of action by which this E3 ligase curbs flavivirus replication.

## Methods

### Plasmids

The retroviral vectors encoding C-terminally Flag-tagged, wild-type (WT) human TRIM56, the E3 Ub ligase-deficient CC21/24AA mutant, and a deletion mutant lacking C-terminal aa 693 to 750, respectively, in the pCX4bsr backbone, have been described previously [25]. N-terminally Flag- and HA-tandem tagged human TRIM56 (FH-T56) was inserted into the pCX4pur retroviral vector backbone, to yield pCX4pur-FH-T56. To express a recombinant TRIM56 protein fragment in *E*. *coli* for polyclonal antibody production and protein-RNA interaction assay, we inserted a cDNA fragment encoding the C-terminal 392 aa of human TRIM56 (T56-C392) into pMAL-c4x (New England Biolabs). The resultant plasmid vector, designated pMAL-c4x-T56-C392, allowed expression and subsequent purification of an MBP-T56-C392 fusion protein in *E*. *coli*. The plasmid encoding serotype 1 DENV replicon, pACYC-DENV1-Rluc2A-Rep [30], was obtained from Ju-Tao Guo and Jinhong Chang (Baruch S. Blumberg Institute) with permission of Pei-Yong Shi (University of Texas Medical Branch). The NS4B coding sequence of ZIKV (MR766 strain) was amplified by PCR from the cDNA of virally infected Vero cells and ligated into pEF6/V5-His-TOPO (Invitrogen) to yield the pEF6-ZIKV-NS4B-V5His6 construct, from which NS4B would be expressed as a protein fused to C-terminal V5-His6 epitope tags. All plasmids were verified by Sanger DNA sequencing [31].

### Cell culture

Human embryonic kidney (HEK) 293, HeLa, SV40 T antigen-transformed human fetal glial cell line SVGA (a gift from Santosh Kumar, University of Tennessee Health Science Center (UTHSC)), human neuroblastoma SK-N-SH (kindly provided by Francesca-Fang Liao, UTHSC), human hepatoma cell lines Huh7 and HLCZ01 (kindly provided by Haizhen Zhu, Hunan University, China) [32], SV40 T antigen-immortalized human hepatocyte PH5CH8, African green monkey kidney cell lines Vero and BSC-1, murine embryonic fibroblasts (MEFs), mouse fibroblast cell line L929, mouse hepatoma Hepa1-6 cells, and mosquito C6/36 cells were maintained in Dulbecco’s modified Eagle medium (DMEM) supplemented with 10% fetal bovine serum (FBS), 100 U/ml of penicillin, and 100 μg/ml of streptomycin. HEK293T and its clonal derivatives deficient in Dicer expression, No-Dice (2–20) and No-Dice (4–25) [33], were kindly provided by Bryan Cullen (Duke University Medical Center).

HeLa-Flp-In T-REx-ACE2 (referred to as HeLa-FitA2) cells with tetracycline (Tet)-inducible expression of 2xHA-tagged, WT, E3 Ub ligase-deficient CC21/24AA mutant, or C-terminal 693–750 aa deletion mutant of TRIM56 have been described in a previous study [34]. Tet-inducible expression of HA-TRIM56-WT had also been established in HEK293-Flp-In T-Rex (referred to as HEK293-FIT, Invitrogen) cells [24]. HEK293-T3Y cells that stably express very low levels of C-terminally YFP-tagged TLR3 were kindly provided by Kate Fitzgerald (University of Massachusetts Medical School). To stably express Flag- and HA-tandem tagged TRIM56 (FH-T56), we transduced HEK293-T3Y cells with a replication-incompetent retrovirus packaged from pCX4pur-FH-TRIM56. Following selection with puromycin, surviving cells were pooled, designated as HEK293-T3Y-FH-T56 cells, and used for subsequent analyses. The blasticidin-resistant, HEK293 cells with stable, constitutive expression of Flag-tagged WT TRIM56 or the E3 ligase-dead CC21/24AA mutant have been described [24]. To create HEK293 cells with constitutive expression of the TRIM56-Flag mutant lacking C-terminal 693–750 residues, cells were transduced with a pCX4bsr-derived retroviral vector carrying the mutant TRIM56 cDNA, followed by stable selection with blasticidin. Similar strategy was adopted to establish SK-N-SH-, HEK293T- and No-Dice (4–25)-derived cell populations with constitutive expression of TRIM56-Flag or the empty pCX4bsr vector, as controls.

Generation of primary mouse neuronal cultures was conducted as follows. A pair of combined cortex and hippocampus with subventricular zone was obtained from E18 C57BL/6 mice (BrainBits, LLC). Neuroprogenitor and differentiated cultures were generated based on established protocols [35, 36]. Specifically, to generate neural progenitor cells (NPCs), tissue was suspended in Dissociation Solution (DS) consisting of Hibernate E-Ca without B27 and Papain (2 mg/ml) (BrainBits, LLC) for 10 min at 30°C, allow the tissue to settle and replace DS with Hibernate EB (HEB) medium (Invitrogen) and triturate with pasture pipette for 1 min and allow undispersed pieces to settle. Supernatant was transferred, centrifuged at 200 x g for 1 min, aspirated and the pellet cells resuspended in NpGrow (BrainBits, LLC). Cells were counted and diluted with NpGrow at 2 ml/10 cm^2^ and plated on ultra-low attachment plates at 2.5 x 10^4^ cells/10 cm^2^ and incubated at 37°C, 5% CO_2_, 95% humidity. Half of the media were exchanged with fresh 37°C CO_2_ equilibrated NpGrow every 3–4 days; neurospheres are present at 4–7 days. To differentiate NPCs into primary neuronal cultures, neurospheres from day 7 cultures were dissociated in DS for 10 min at 30°C, centrifuged at 200 x g, and DS was replaced with NbActiv4 neuron culture medium (BrainBits). Cells were counted and seeded into 24-well plates at 2 x 10^5^ cells/well and incubated at 37°C, 5% CO_2_, 95% humidity. Half of the media were exchanged with fresh NbActiv4 media every 3–4 days; axons and dendrites are observable by day 4 while synapses and action potentials have been reported at 7 days [35, 36].

### Viruses, infection of cultures, and viral titer assay

ZIKV MR766 strain (BEI Resources # NR-50065) and PRVABC59 strain (kindly provided by Brandy Russel, CDC, Fort Collins, CO) were propagated in C6/36 cells. For infections of various cell types, cells were inoculated with ZIKV in 2% FBS-containing DMEM at indicated multiplicities of infection (MOIs) for 1–2 h. Subsequently, viral inoculum was removed, and cells were washed three times in PBS, refed with complete culture medium and cultured for the indicated time periods. Infectious virus titers in cell-free culture supernatants were determined by an endpoint dilution-based 50% tissue culture infective dose (TCID_50_) assay in 96-well plates [37, 38]. Titration of ZIKV was performed on Vero cells, and cytopathic effect (CPE) was recorded and used for calculation of viral titers at 7 days post infection (dpi) by the Reed and Muench method [39].

Infections of primary mouse neuronal cultures were carried out at day 8 post-differentiation when medium was removed and the cells were washed twice with HBSS. Based on cell counts from 3 wells, neurons were either mock -infected (controls) or infected with ZIKV-PRVABC59 at a MOI of 1 in NbActiv4 media for 2 h at 37°C; duplicate wells for each time point were used. Inoculum was then removed, cells washed twice with NbActiv4, and then incubated in 0.5 ml of NbActiv4 media at 37°C, 5% CO_2_, 95% humidity. At 24 and 48 h after infection, 0.5 ml TRIzol was added to each well for RNA extraction and qPCR analysis.

### RNA interference (RNAi)

HeLa cells with stable, shRNA-mediated knockdown of TRIM56 (HeLa-shT56) and control cells stably transduced with a non-targeting shRNA (HeLa-shCtrl) were described in a previous study [24]. To deplete TRIM56 expression in SVGA cells, we transduced the cells with lentiviral shRNA-094 specifically targeting human TRIM56 [24] and selected cell populations resistant to puromycin. For comparison, control cells were generated by transduction with a non-targeting control lentiviral shRNA packaged from pLKO.1-shRNA-scramble (Addgene# 1864), followed by selection in puromycin-containing medium. For transient knockdown of TLR3, a synthetic siRNA specifically targeting human TLR3 (siTLR3) [40] was transfected into cells by Lipofectamine 2000 for the indicated times as per manufacturer’s instructions (Invitrogen). As a control for comparison, a non-targeting negative control siRNA (Invitrogen AM4636) was used in lieu of siTLR3.

### Quantitative PCR

Extraction of total cellular RNA by TRIzol (Invitrogen), cDNA synthesis by reverse transcription, and quantitative PCR (qPCR) were implemented as described elsewhere [25, 41]. The following primers were used to detect ZIKV RNA (specifically recognizing the NS4B-coding region): ZIKV-4B-7260F, 5’-GCACTACATGTACTTGATC-3’; and ZIKV-4B-7367R, 5’-ACCACTATTCCATCCACAAC-3’. Primers specific for human innate immune genes including *TLR3*, *IFNB*, *IL29*, *RANTES* and *ISG56* have been described [34, 42]. The relative abundance of each target was normalized to that of *28S* rRNA [34]. Copy numbers of ZIKV RNA were calculated based on standard curves generated using serially diluted pEF6-ZIKV-NS4B-V5His6 DNA that ranged from 10^4^ to 10^7^ copies/ml.

For miRNA detection, we synthesized oligonucleotide primers and adopted the protocol as described in the qSTAR miRNA qPCR detection system (Origene). In brief, after a poly(A) tailing procedure, a miR-oligo-dT primer with a sequence of 5’-GAACATGTCTGCGTATCTCAGACTTCTGATTCACGCTTTTTTTTTTTTTTTTTTTVN-3’, was used for reverse transcription of total small RNAs. Subsequently, SYBR green-based qPCR was performed to measure the levels of miRNAs of interest relative to that of an internal control, *i*.*e*., U6 snRNA. The primers for qPCR detection included a miRNA/snRNA-specific forward primer and a universal reverse primer, as follows. miR-92a, 5’-TATTGCACTTGTCCCGGC-3’ (forward); miR-21, 5’-TAGCTTATCAGACTGATGTTG-3’ (forward); U6 snRNA, 5’-CTGCGCAAGGATGACACGC AA-3’ (forward); and miR-Rev-universal, 5’-GAACATGTCTGCGTATCTC-3’ (reverse).

### Protein analyses

Cell lysates were prepared in RIPA buffer and subject to SDS-PAGE and immunoblotting analysis were described previously [25, 43]. Immunofluorescence staining were performed as previously described [25, 43]. The following monoclonal (mAb) and polyclonal (pAb) antibodies were utilized: mouse anti-Flag-tag M2 mAb (Sigma); mouse anti-HA-tag mAb (Invivogen); rabbit anti-maltose-binding protein (MBP) pAb (New England Biolabs); mouse anti-flavivirus envelope protein (4G2) mAb (Millipore); mouse anti-ZIKV NS5 mAb, clone 8B8 (Biofront Technologies, kindly provided by Hengli Tang, Florida State University); mouse anti-β-actin mAb (Sigma); rabbit anti-TRIM56 pAb (Bethyl Labs) or rabbit anti-TRIM56 S4091 pAb (generated by immunizing rabbits at Proteintech Group Inc. with a recombinant protein comprising the C-terminal 392 aa of human TRIM56 fused to MBP that was expressed and purified from *E*. *coli*); peroxidase-conjugated secondary goat anti-rabbit and goat anti-mouse pAbs (Southern Biotech); FITC-conjugated secondary goat anti-mouse pAb (Southern Biotech). Specifically, FH-T56 was detected by mouse anti-Flag-tag M2 mAb (Sigma); rabbit anti-TRIM56 S4091 pAb was used to detect endogenous T56 protein in HeLa cell lysates; and rabbit anti-TRIM56 pAb (Bethyl Labs) was used for other T56 immunoblotting experiments.

### Ribonucleoprotein (RNP) immunoprecipitation (RNP-IP)

ZIKV-infected HEK293 cells expressing control vector, or Flag-tagged, WT or mutant TRIM56, respectively, were washed twice with ice-cold PBS and lysed in a buffer containing 50 mM Tris-HCl (pH 8.0), 150 mM NaCl, 1% NP-40, 0.25% sodium deoxycholate, and protease inhibitor cocktail (Sigma). The cell lysates were incubated at 4°C for 30 min, clarified by centrifugation at 12, 000 g at 4°C for 15 min, and their protein concentrations were adjusted to 1 μg/μl. For RNP-IP, 400 μg of cell lysates were incubated with the anti-Flag mAb at 1:500 at 4°C overnight. Subsequently, RNA-protein-antibody complexes were captured by incubation with protein A/G agarose beads (Santa Cruz Biotech). After three washes, the beads were divided equally into two fractions, which were subjected to RNA isolation by TRIzol and protein extraction by boiling in SDS-sample buffer, respectively. The qPCR analysis of ZIKV RNA levels and immunoblotting of TRIM56 were performed as described above.

### Cell-free assay for TRIM56-ZIKV RNA interaction

To assess whether TRIM56 directly interacts with ZIKV RNA, we first purified MBP-tagged T56-C392 (comprising the C-terminal 392 aa of human TRIM56) protein from *E*. *coli* using the pMAL Protein Fusion and Purification System (New England Biolabs, E8000S, USA). To produce a control protein, we purified MBP along with a short stretch of the downstream polylinker (MBP-polylinker) from *E*. *coli* transformed with the empty vector pMAL-c4x. ZIKV RNA was extracted using TRIzol from virions in high-titer ZIKV stocks. Subsequently, two micrograms of MBP-polylinker or MBP-T56-C392 protein were incubated with 0.5 μg of ZIKV RNA at room temperature for 30 min, followed by pull-down of MBP-tagged proteins using amylose resin. After three washes, the resins were divided equally into two fractions, which were subjected to RNA isolation by TRIzol and protein extraction by boiling in SDS sample buffer, respectively. Quantification of ZIKV RNA levels was performed by qPCR as described above. MBP-polylinker and MBP-T56-C392 in the protein samples were probed by immunoblotting using rabbit anti-MBP pAb (New England Biolabs) and rabbit anti-TRIM56 pAb (Bethyl Labs).

### Statistical analysis

All results are presented as means ± standard deviations. For analysis of statistical differences, two-tailed student t-test (Excel 2016, Microsoft, USA) was used to compare the means of two groups and one-way ANOVA followed by Tukey or Dunnett test (GraphPad Prism 5.0, USA) was applied for comparing the means of more than two groups. Differences with a *P* value of < 0.05 were deemed statistically significant.

## Results

### Broad tropism of ZIKV in cell culture

To study host factors that impact ZIKV replication, we first surveyed a number of human and animal cell lines of various tissue origins for ZIKV susceptibility. Cells were infected by MR766, a prototype African strain of ZIKV, followed by immunofluorescence staining of viral E protein expression at 72 h.p.i. Three human hepatocyte cell lines, including Huh7, PH5CH8, and HLCZ01, were all susceptible to ZIKV infection, albeit to different degrees (S1 Fig). The hepatoma Huh7 cells were previously reported to support ZIKV infection [44]. We found Huh7 cells supported the highest efficiency of ZIKV infection, with 100% of cells infected and exhibiting significant cytopathic effect (CPE) at 72 h.p.i. HLCZ01, a recently established human hepatoma line, was also highly susceptible, with >90% cells infected by ZIKV. In comparison, PH5CH8, a non-neoplastic hepatocyte line that harbors intact innate immune responses resembling primary human hepatocytes [38, 40, 45], was permissive for ZIKV but less susceptible than Huh7 and HLCZ01. HeLa-FitA2 cells, which were derived from the human cervical epithelial carcinoma cell line HeLa and stably express the Tet repressor and harbors a single integrated Flp recombination target site allowing for rapid generation of Tet-inducible stable cell lines, also supported robust ZIKV infection (S2 Fig). The human embryonic kidney HEK293 cells had been reported to support low efficiency of ZIKV infection [11]. We examined three different HEK293-derivatives, HEK293-FIT, HEK293-T3Y and 293T cells, and found they supported varying degrees of ZIKV replication (see below). A notable feature of ZIKV infection in humans is its neurotropism [46–48]. We thus examined two human cell lines of neural origin, *i*.*e*., the neuroblastoma line SK-N-SH and SVGA, a fetal glial cell line, and found both to be permissive for ZIKV infection (S3 Fig). Apart from the human cell lines, we found the African green monkey kidney cell line BSC1 and the Madin–Darby canine kidney (MDCK) cell line both to be susceptible to ZIKV infection (S4 Fig). We also examined the susceptibility of three commonly used mouse cell lines, Hepa1-6, L929 and MEF, to ZIKV infection and compare these lines with Huh7 and HEK293, which represent human cell lines with relatively high and low permissiveness for ZIKV infection, respectively. Cells were infected with ZIKV-MR766 for different time periods, followed by quantification of intracellular viral RNA abundance by qPCR that sensitively detects viral RNA replication. As shown in S5 Fig, Huh7 cells were highly permissive, harboring 10^8^ copies of ZIKV RNA per microgram of total RNA already at 24 h.p.i. There was a further, ~4-fold increase in viral RNA levels at 48 h.p.i., which reached sub-10^9^ copies/μg total RNA range. In contrast, HEK293 cells were much less permissive, harboring mid-10^6^ copies/μg total RNA range of viral RNAs at 24 h.p.i. ZIKV RNA replication increased by ~18-fold in HEK293 cells at 48 h.p.i, approaching that of Huh7 cells at 24 h.p.i. However, all three murine cell lines examined (hepa1-6, L929 and MEF) exhibited limited permissiveness, if any, for ZIKV, harboring mid-10^5^ to ~10^6^ copies/μg total RNA range viral RNA at 24 h.p.i. Moreover, viral RNA levels did not increase in hepa1-6 and MEFs, while had a mere ~2-fold uptick in L929 cells, at 48 h.p.i. Collectively, these data demonstrate that ZIKV infects and replicates in a broad range of human and animal cell lines, although the susceptibility of different cell lines vary considerably.

### Ectopic expression of TRIM56 inhibits propagation of ZIKV

**Fig 1:**
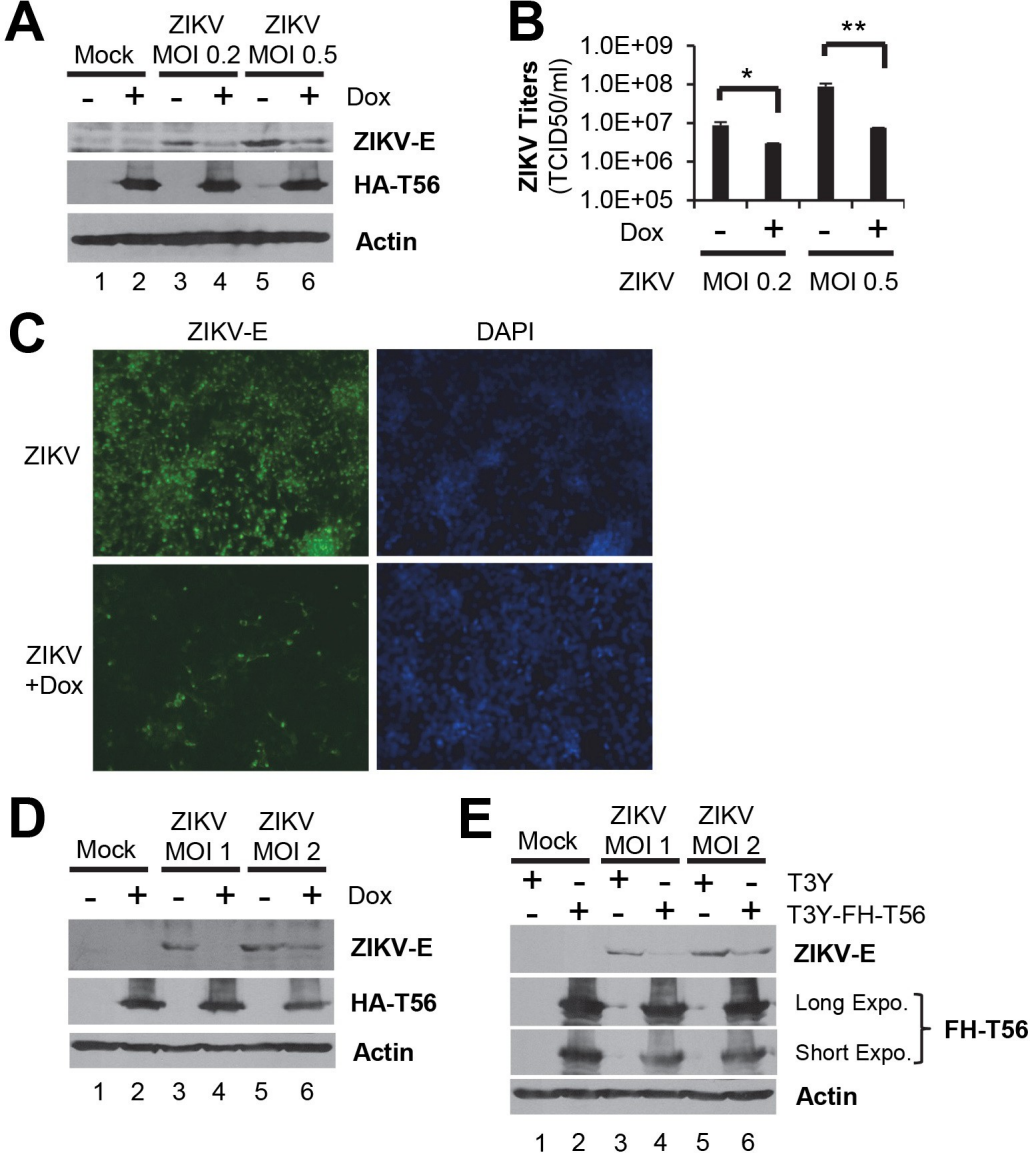
Ectopic expression of TRIM56 inhibits ZIKV-MR766 infection. (A) HeLa-FitA2-T56 cells were pretreated with or without 1 μg/ml Dox for 36 h, followed by infection by ZIKV MR766 strain at indicated MOIs in the presence or absence of Dox for 72 h. The expression of ZIKV E, HA-TRIM56 and β-actin (loading control) proteins was determined by immunoblotting. T56, TRIM56. (B) HeLa-FitA2-T56 cells were manipulated as described in (A) and the progeny ZIKV yields in culture supernatants were determined by TCID_50_ assay. Student t-test, *P<0.05, **P<0.01. Results were representative of three independent experiments. (C) Immunostaining of ZIKV E protein (green) in HeLa-FitA2-T56 cells with or without HA-TRIM56 induction by Dox treatment and infected with ZIKV MR766 strain (MOI = 0.5). DAPI was used for counterstaining of nuclei (blue). Images were representative of two independent experiments. (D) HEK293-Fit-T56 cells were manipulated as described for HeLa-FitA2-T56 cells in (A), followed by immunoblotting of ZIKV E, HA-TRIM56, and β-actin (loading control) proteins. (E) HEK293-T3Y cells with or without expression of FH-TRIM56 were infected with ZIKV MR766 strain for 72 h, followed by immunoblot analysis of ZIKV E, FH-TRIM56, and β-actin (loading control) proteins. FH-T56, Flag-HA-TRIM56. Immunoblots in panels A, D, and E were representative of at least three independent experiments.

Our previous studies have shown that TRIM56 is an antiviral host factor against several different RNA viruses, among which include 3 members of the Flaviviridae, DENV2, YFV, and BVDV [24, 25]. To determine whether TRIM56 impacts ZIKV fitness, we conducted infection experiments in HeLa-FitA2-T56 cells with Tet-inducible expression of HA-tagged TRIM56 we developed previously [34]. In these cells robust expression of HA-TRIM56 protein could be turned on upon addition of doxycycline (Dox) to culture medium (Fig 1A, compare lanes 2 vs l). When infected with ZIKV-MR766, cells without HA-TRIM56 expression harbored abundant viral E protein at 72 h.p.i. (Fig 1A, lanes 3 and 5). In contrast, the level of ZIKV E protein was profoundly reduced in cells with Dox-induced HA-TRIM56 expression (Fig 1A, lanes 4 and 6). Consistent with the viral protein data, progeny virus titers in culture supernatants were significantly lower in cells induced for HA-TRIM56 expression than those cultured in the absence of Dox (Fig 1B, compare bars 4 vs 3, a 11.7-fold decrease at MOI 0.5). Immunofluorescence staining revealed that the percentage of cells positive for ZIKV E antigen was substantially lower in cells with Dox treatment than those without (Fig 1C). To ensure this is not a cell-type-specific phenomenon, we assessed the anti-ZIKV activity of TRIM56 in HEK293-FIT-T56 cells that express HA-TRIM56 in a Tet/Dox-inducible manner [24]. As shown in Fig 1D, the results mirrored those obtained in HeLa-FitA2-T56 cells. In addition to the Tet-inducible expression system, a constitutive overexpression strategy was also utilized to examine the impact of TRIM56 on ZIKV infection. Retrovirus-mediated ectopic expression of Flag-HA-tagged TRIM56 (FH-T56) curtailed viral E protein expression in HEK293-T3Y cells infected by ZIKV (Fig 1E, compare lanes 4 vs 3, and lanes 6 vs 5). Taken together, these results reveal that ectopic expression of TRIM56 inhibits the propagation of an African strain of ZIKV.

### TRIM56 hinders infection by an Asian lineage ZIKV

**Fig 2:**
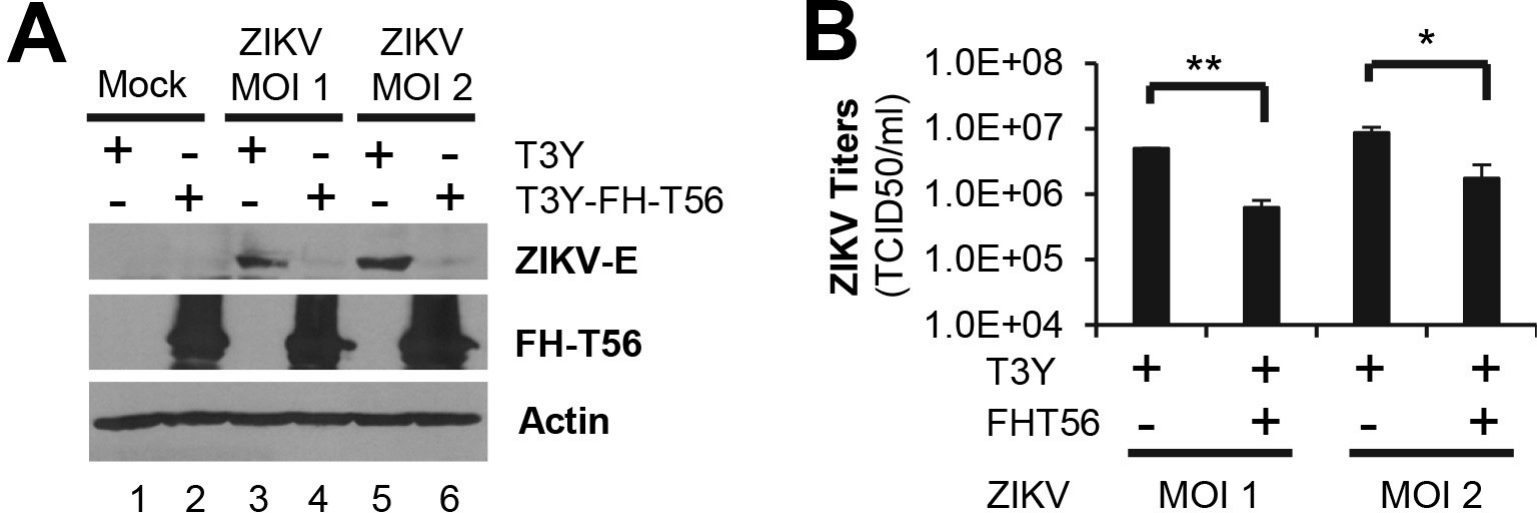
TRIM56 suppresses propagation of an Asian lineage ZIKV. (A and B) HEK293-T3Y cells with or without expression of FH-TRIM56 were infected with ZIKV PRVABC59 strain at indicated MOIs for 72 h. Intracellular viral E protein (A) and extracellular progeny virus yields (B) were analyzed by immunoblotting and TCID_50_ assay, respectively. Results shown in bar graphs were analyzed by Student t-test and were reproduced twice, *P<0.05, **P<0.01. The immunoblotting data were representative of two independent experiments.

Since the recent ZIKV outbreaks were caused by viruses of Asian lineage, we sought to verify the anti-ZIKV effect of TRIM56 using the PRVABC59 strain, an Asian lineage ZIKV [49]. When challenged with this virus, control cells without FH-TRIM56 expression supported abundant expression of ZIKV E protein at 72 h.p.i. (Fig 2A, lanes 3 and 5), whereas cells constitutively expressing FH-TRIM56 had diminished viral protein levels (lanes 4 and 6), even when infected at a high MOI (MOI = 2, compare lanes 6 vs 5). Data on progeny virus production in culture supernatants (Fig 2B) also confirmed the inhibitory effect of TRIM56 on ZIKV-PRVABC59 propagation.

### The endogenous TRIM56 protein restricts ZIKV infection

**Fig 3:**
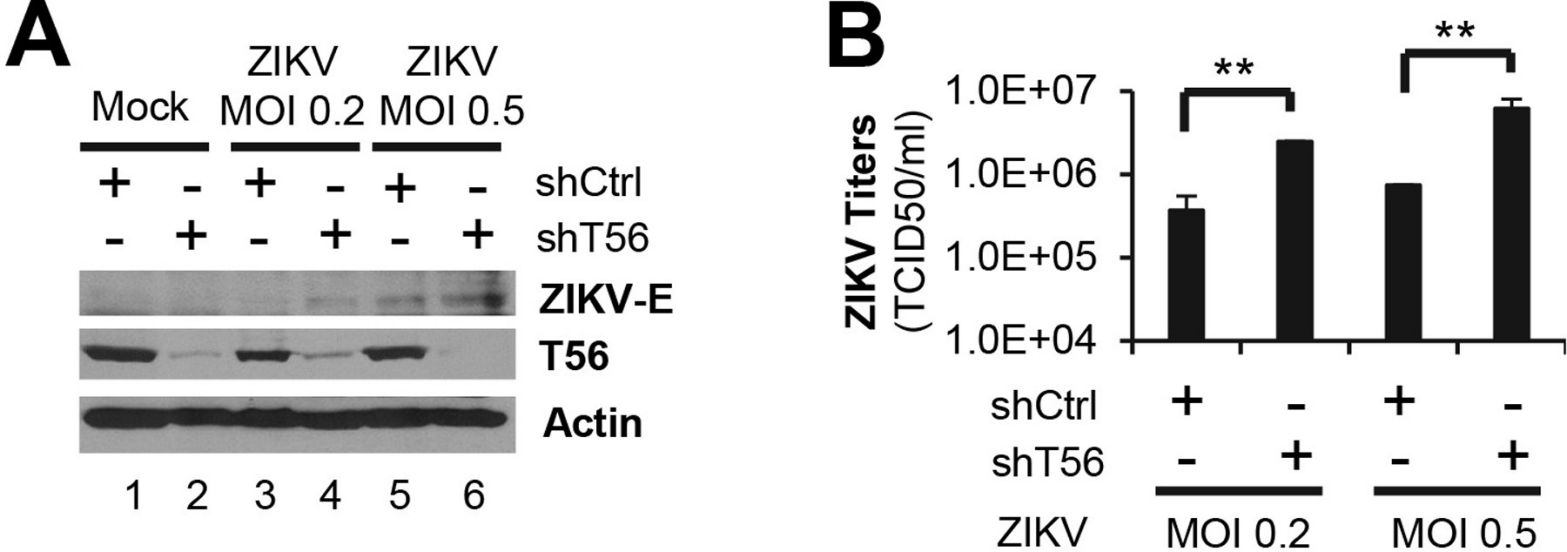
Depletion of TRIM56 facilitates ZIKV infection. (A) HeLa cells stably transduced with a non-targeting control shRNA (shCtrl) or TRIM56-specific shRNA (shT56) were infected by ZIKV MR766 strain at indicated MOIs for 72 h. ZIKV E and TRIM56 proteins were analyzed by immunoblotting. β-actin was used as a loading control. Data were representative of three independent experiments. (B) HeLa-shCtrl and HeLa-shT56 cells were infected by ZIKV MR766 strain as described in (A) and progeny virus production in culture supernatants was measured by TCID_50_ assay. Student t-test, **P<0.01. Results were representative of three independent experiments.

To determine whether TRIM56 expressed at physiologic levels impedes propagation of ZIKV, we depleted endogenous TRIM56 in HeLa cells by shRNA-mediated knockdown and evaluated the changes in viral protein expression and progeny virus production. As shown in Fig 3A, the abundance of ZIKV E protein was higher in cells with efficient TRIM56 knockdown (shT56) than in cells bearing a nontargeting, scrambled control shRNA (shCtrl) (compare lanes 4 vs 3, and lanes 6 vs 5). Moreover, shT56 cells consistently yielded ~ 1-log more progeny virus than did shCtrl cells, when infected at two different MOIs (Fig 3B). These data establish TRIM56 as a restriction factor of ZIKV when expressed at physiologically relevant levels.

### The E3 ligase activity and C-terminal part are both pivotal for TRIM56-mediated restriction of ZIKV

**Fig 4:**
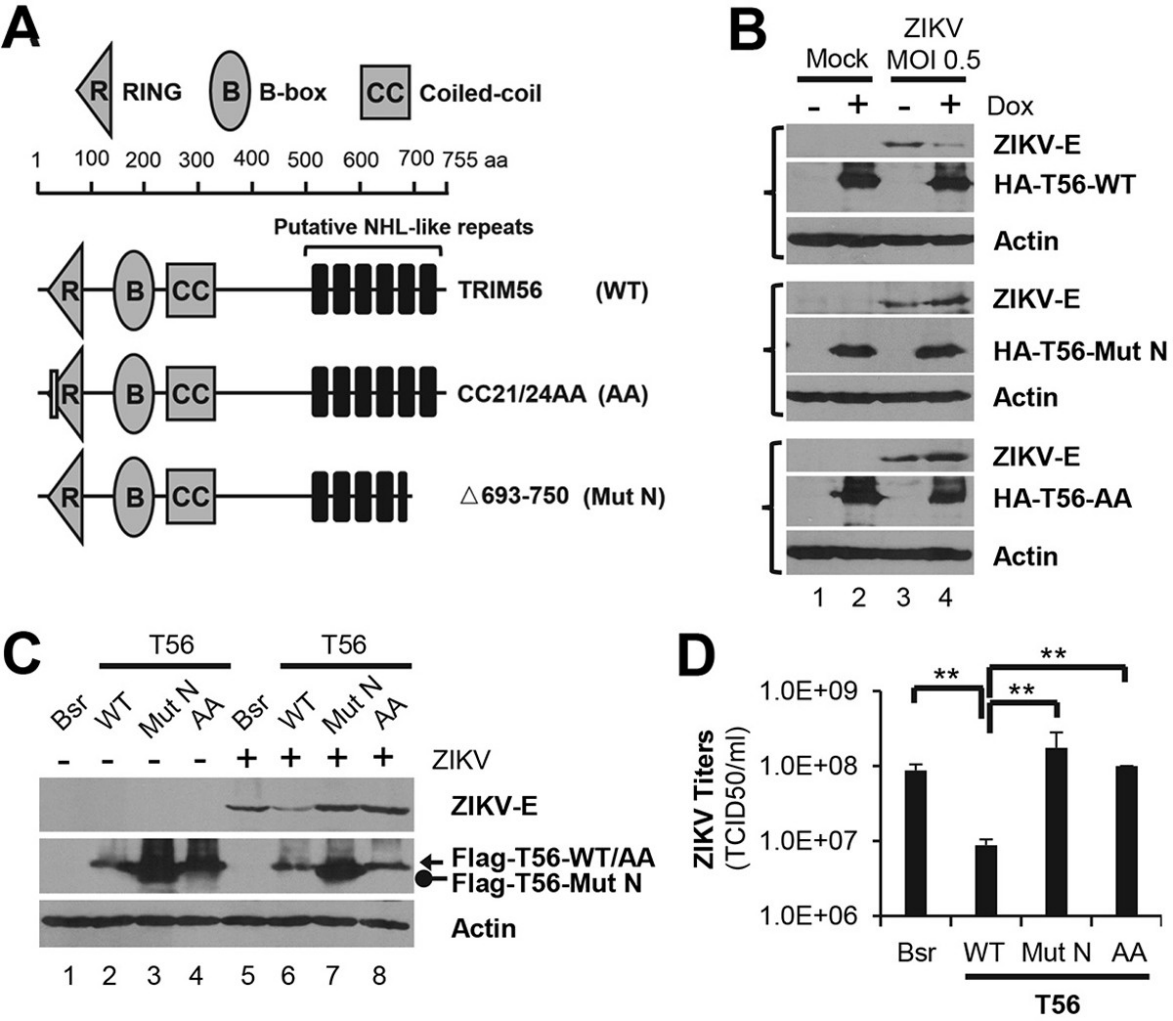
The E3 ligase activity and C-terminal portion are both critical for the anti-ZIKV action of TRIM56. (A) Schematic representation of TRIM56 domains and the individual TRIM56 mutants investigated in this study. (B) HeLa-FitA2 cells with Dox-inducible expression of HA-T56-WT, HA-T56-Mut N, or HA-T56-AA were infected by ZIKV MR766 strain (MOI = 0.5) in the presence or absence of Dox for 72 h. The expression of ZIKV E, actin (loading control), and HA-tagged TRIM56 proteins were determined by immunoblotting. (C and D) HEK293 cells stably transduced with empty vector (Bsr), or Flag-T56-WT, Flag-T56-Mut N or Flag-T56-AA were infected by ZIKV MR766 strain for 72 h. Intracellular expressions of ZIKV E, actin (loading control), and Flag-tagged TRIM56 proteins were determined by immunoblotting (C), and progeny virus production in culture supernatants was measured by TCID_50_ assay (D). Results shown in bar graphs were analyzed by one-way ANOVA test and reproduced for three times, **P<0.01. The immunoblotting data were representative of three independent experiments.

To understand how TRIM56 exerts its antiviral action, we determined the domains or activities that are critical for TRIM56 to restrict ZIKV infection. The main functional domains of TRIM56 and its various mutants are illustrated in Fig 4A. We first utilized three cell lines derived from HeLa-FitA2 that stably express, in a Dox-inducible fashion, HA-tagged wildtype TRIM56 (WT), the E3 Ub ligase-deficient CC21/24AA mutant (AA), and the C-terminal aa 693–750 deletion mutant (Mut N), respectively [34]. As shown in Fig 4B, comparable expression of WT TRIM56 and mutant proteins was achieved following Dox treatment. When challenged with ZIKV (MOI = 0.5), cells in which WT TRIM56 was induced harbored substantially lower level of ZIKV E protein than cells without Dox induction (Fig 4B, upper panels, compare lanes 4 vs 3). In contrast, intracellular ZIKV E protein abundance was unaffected following Dox induction of either the E3 Ub ligase-dead AA mutant or the C-terminal deletion mutant (Mut N) (Fig 4B, middle and lower panels).

To corroborate this finding, we evaluated ZIKV propagation efficiency in HEK293 cells stably transduced with an empty retroviral vector (Bsr), WT or TRIM56 mutants (AA and Mut N), respectively. Compared with that in Bsr control cells, ZIKV E protein abundance was reduced in cells overexpressing WT TRIM56 (Fig 4C, compare lanes 6 vs 5) but not in cells overexpressing either TRIM56 mutant (Fig 4C, compare lanes 7 and 8 vs 5). The AA and C-terminal aa 693–750 deletion mutants also lost their abilities to limit progeny ZIKV production, as compared with WT TRIM56 (Fig 4D, compare bars 3 and 4 vs 2, increases of ~12-19-fold). In aggregate, these results suggest that the E3 Ub ligase activity and the C-terminal 693-750aa portion are both indispensable for TRIM56-mediated restriction of ZIKV.

### TRIM56 suppresses ZIKV RNA replication

**Fig 5:**
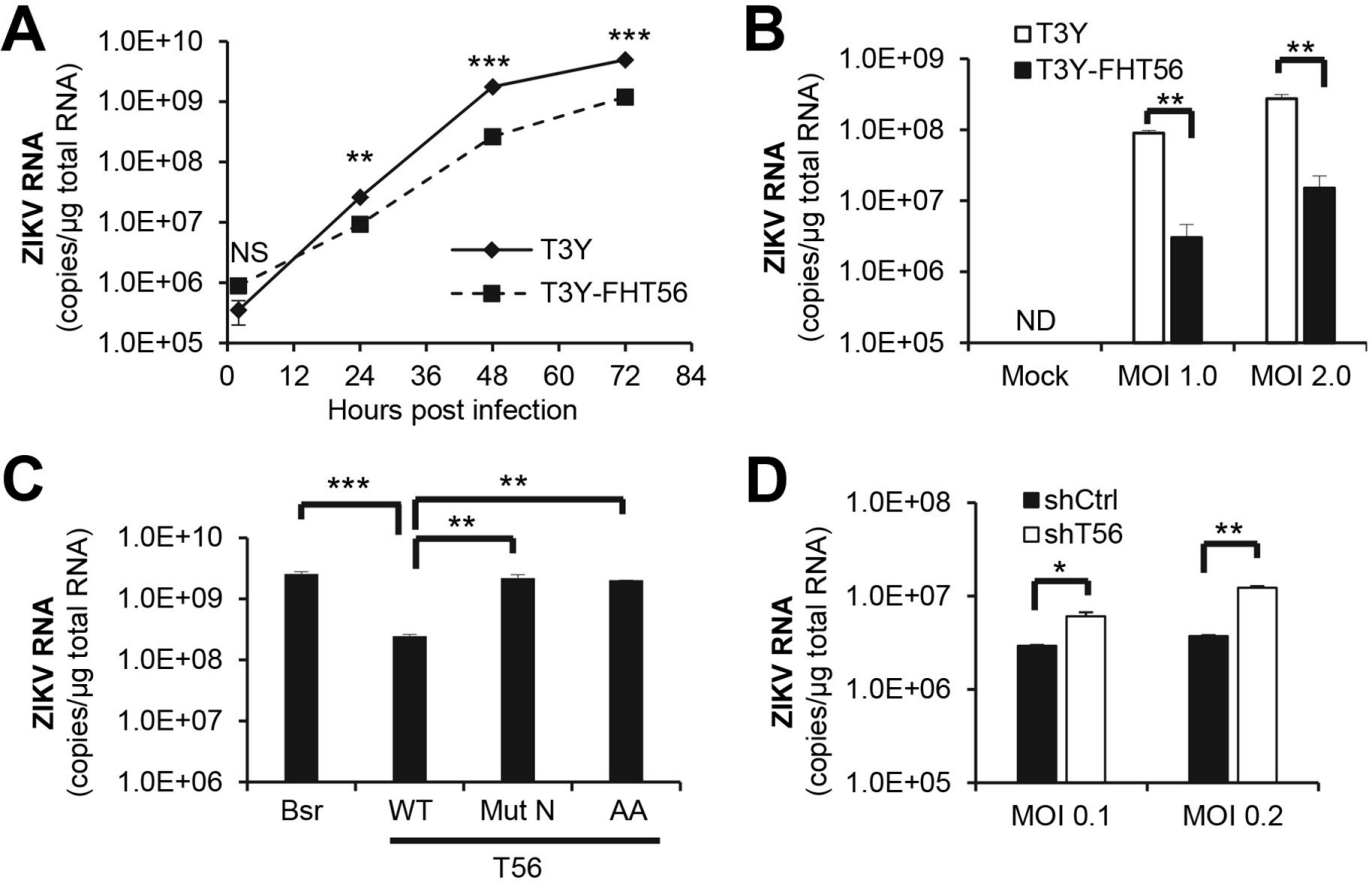
TRIM56 inhibits ZIKV RNA replication. (A) HEK293-T3Y cells with or without expression of FH-TRIM56 were infected by ZIKV MR766 strain (MOI = 1) for the indicated times, followed by qPCR analysis of intracellular viral RNA levels. NS, not significant. (B) Intracellular viral RNA levels in HEK293-T3Y cells with or without expression of FH-TRIM56 that were infected by ZIKV PRVABC59 strain at indicated MOIs for 72 h. ND, non-detectable. (C) Intracellular viral RNA levels in HEK293 cells stably transduced with empty vector (Bsr), Flag-T56-WT, Flag-T56-Mut N, or Flag-T56-AA that were infected by ZIKV MR766 strain (MOI = 1) for 72 h. (D) Intracellular viral RNA levels in HeLa-shCtrl and HeLa-shT56 cells infected by ZIKV MR766 strain for 72 h. Results shown in panel B were representative of two independent experiments. Results in the rest panels were representative of three independent experiments. One-way ANOVA test (in panel C) or Student t-test (in the rest panels), *P<0.05, **P<0.01, ***P<0.001.

TRIM56 targets certain RNA viruses for inhibition at different steps of viral life cycle. It inhibits influenza viruses, YFV and DENV2 by impeding viral RNA synthesis [24, 26] but curbs human coronavirus (HCoV)-OC43 by acting at the stage of viral packaging/release [24]. To pinpoint where TRIM56 exerts its antiviral action during ZIKV life cycle, we compared the kinetics of intracellular viral RNA accumulation at different times following infection between control and TRIM56-overexpressing cells. At 2 h.p.i., a time point immediately after viral entry and prior to initiation of viral RNA replication, intracellular viral RNA levels were comparable between control HEK293-T3Y cells and cells expressing FH-T56, suggesting that TRIM56 does not affect cellular entry of ZIKV (Fig 5A). Intracellular viral RNA abundance began to climb at 24 h.p.i. in both cells but did so much more quickly and robustly in cells without FH-T56 expression. For all three time points examined at/after 24 h.p.i., ZIKV RNA replicated to significantly lower levels in FH-T56 cells than in control HEK293-T3Y cells (Fig 5A). Confirming the effect was not virus strain-specific, we obtained similar results from experiments using ZIKV-PRVABC59 in lieu of ZIKV-MR766 (Fig 5B). Importantly, the inhibitory effect of TRIM56 on viral RNA replication was lost upon mutations that abolish the E3 ligase activity or delete its C-terminal portion (Fig 5C). Furthermore, ZIKV RNAs replicated to significantly higher levels in HeLa-shT56 cells with stable TRIM56 knockdown than in HeLa-shCtrl cells (Fig 5D), suggesting that endogenous TRIM56 shares the effect with overexpressed TRIM56. We conclude from these data that TRIM56 restricts ZIKV infection by inhibiting viral RNA replication, and that such capacity depends on both its E3 ligase activity and the integrity of C-terminal portion. Of note, these observations are reminiscent of the effects of TRIM56 on BVDV, YFV, and DENV2 [24, 25], suggesting a shared antiviral mechanism against flaviviruses. To determine whether this can be said with another flavivirus, we electroporated a luciferase-encoding DENV1 replicon into HEK293-FIT-T56 cells that were repressed (-Dox) or induced (+Dox) for HA-TRIM56 expression and followed up the viral RNA replication kinetics (S6 Fig). Similar to the effects of TRIM56 on BVDV and DENV2 replicons [24, 25], the result showed that while translation of the input DENV1 replicon RNA (at 3 h post electroporation) was not affected by TRIM56, viral RNA replication at later time points was always significantly lower in cells with HA-TRIM56 induction than those not induced.

### The inhibition of ZIKV replication by TRIM56 is independent of the biogenesis of or regulation by host miRNAs

Data from the current study on ZIKV as well as from several others on BVDV, YFV, DENV2 and influenza viruses, all underscore the importance of the C-terminal portion of TRIM56 for restricting RNA viruses [24–26]. While TRIM56 is classified within the subgroup C-V that consists of TRIM proteins without known functional domains in their C-terminal regions, the C-terminal portion of TRIM56 exhibits sequence homology with the NHL repeat of several TRIM-NHL proteins including TRIM2, TRIM3, TRIM32 and TRIM71 [26]. Of these, TRIM71 and TRIM32 were reported to bind to miRNAs and/or mRNAs and regulate their function or metabolism [27–29]. Thus, we tested the possibility that the NHL-like repeat in the C-terminal portion of TRIM56 may contribute to antiviral activity via a miRNA-dependent mechanism.

**Fig 6:**
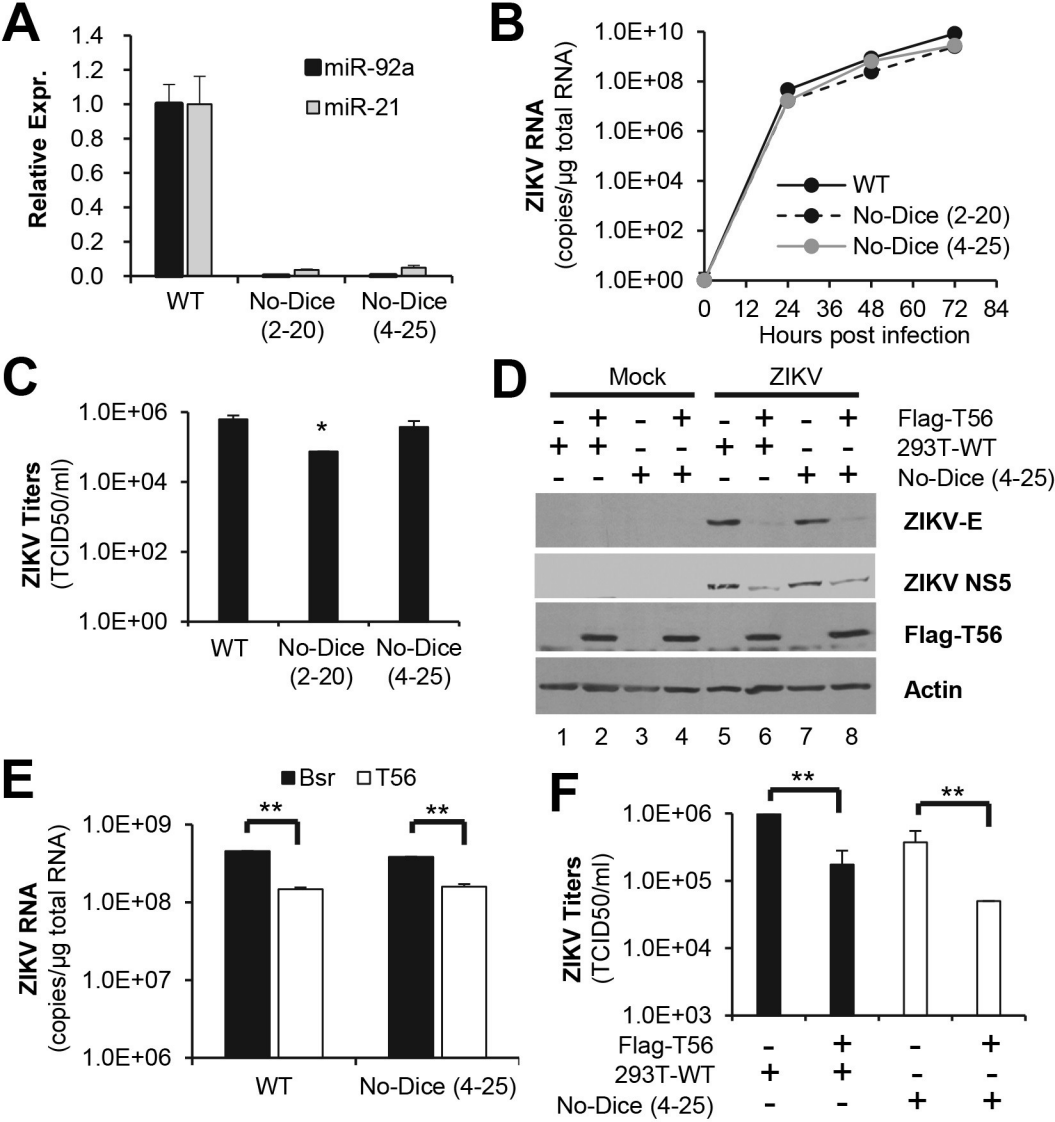
The inhibition of ZIKV infection by TRIM56 is independent of the biogenesis of or regulation by host miRNAs. (A) Expression of miR-92a and miR-21 in control HEK293T cells (WT) and two Dicer-knockout clonal cell lines (No-Dice (2–20) and (4–25)) was determined by qPCR and normalized to U6 snRNA. Results were representative of two independent experiments. (B and C) WT HEK293T and No-Dice cells were infected by ZIKV MR766 strain (MOI = 1) for indicated time points, followed by qPCR analysis of the kinetics of intracellular ZIKV RNA replication (B) and TCID_50_ assay of progeny virus production in culture supernatants at 72 h.p.i. (C). Student t-test, *P<0.05. Results were reproduced three times. (D-F) WT HEK293T and No-Dice (4–25) cells were stably transduced with empty vector (Bsr) or Flag-T56 and subsequently infected by ZIKV MR766 strain (MOI = 1) for 72 h. Intracellular expression of viral E and NS5 proteins, action, and Flag-T56 were analyzed by immunoblotting (D), and intracellular ZIKV RNA levels was quantified by qPCR (E). Student t-test, **P<0.01. Progeny virus production in culture supernatants was measured by TCID_50_ assay (F). Student t-test, **P<0.01. Results in panels D to F were reproduced three times.

To this end, we investigated the impact of TRIM56 on ZIKV infection in 293T-derived cells deficient in Dicer expression thus lacking the biogenesis of miRNAs [33]. First, we confirmed the Dicer deficiency phenotype in two clonal cell lines (referred to as No-Dice (2–20) and (4–25)) by showing the absence of miR-92a and miR-21 expression, as opposed to control 293T (referred to as 293T-WT) cells (Fig 6A). It should be noted that total small RNA deep sequencing analysis had shown these two Dicer-knockout 293T cell lines were deficient in miRNA expression globally, as opposed to 293T-WT cells, in a previous study [33]. Our miRNA qPCR data thus confirmed the reported phenotype of these cells. Next, we wondered if Dicer deficiency affected ZIKV fitness in these 293T-derived cells. Although No-Dice (2–20) cells were slightly impaired for supporting intracellular ZIKV RNA replication (Fig 6B) and progeny virus production (Fig 6C), No-Dice (4–25) cells permitted ZIKV propagation at efficiencies comparable to 293T-WT cells (Fig 6B and 6C). These data suggest that host miRNAs are not essential for ZIKV replication. In subsequent experiments, we utilized No-Dice (4–25) and 293T-WT cells for comparison to dissect the possible role of host miRNAs in the anti-ZIKV action of TRIM56.

Ectopic expression of Flag-tagged TRIM56 or empty control vector was achieved in 293T-WT and No-Dice (4–25) cells by retroviral-mediated gene transfer, and there was comparable expression of Flag-TRIM56 between the two cell lines (Fig 6D, compare lanes 4 vs 2). Following ZIKV infection, profound reductions in ZIKV E and NS5 protein levels were observed in TRIM56-overexpressing cells as compared with control vector-transduced cells, irrespective of Dicer deficiency (Fig 6D). The same could be said of intracellular viral RNA replication (Fig 6E) or progeny virus production (Fig 6F). Collectively, these data show that the deficiency of Dicer does not undermine the inhibitory effect of TRIM56 on ZIKV infection, suggesting an antiviral mechanism independent of the biogenesis of or regulation by host miRNAs.

### TRIM56 associates with ZIKV RNA via its C-terminal portion

**Fig 7:**
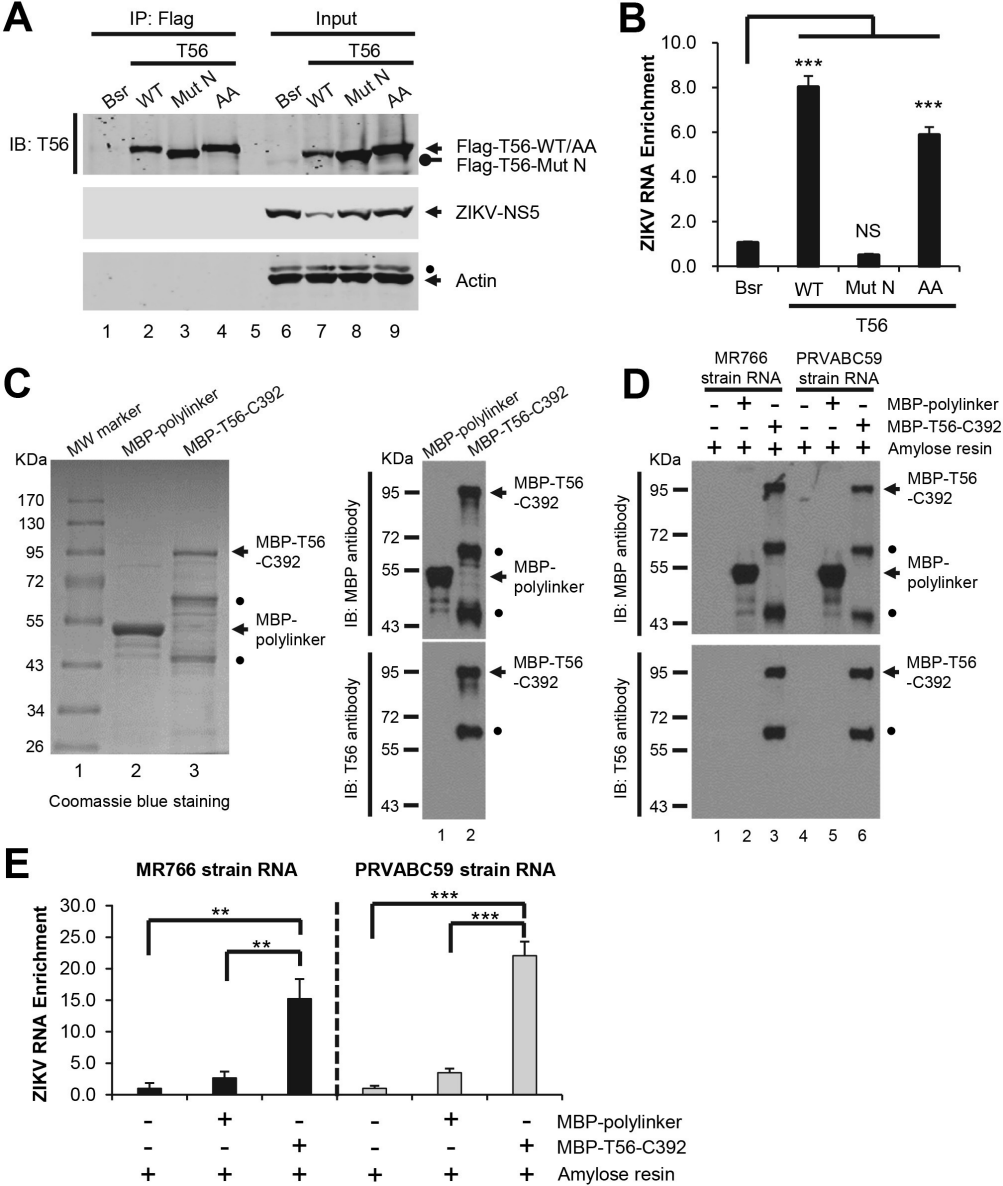
TRIM56 associates with ZIKV RNA in virus-infected cells and in cell-free reactions via its C-terminal portion. (A and B) HEK293 cells stably transduced with empty vector (Bsr), Flag-T56-WT, Flag-T56-Mut N, or Flag-T56-AA were infected by ZIKV MR766 strain (MOI = 1) for 72 h, and TRIM56-RNA complex was immunoprecipitated from whole cell lysates using anti-Flag antibody. Immunoblot analysis of Flag-TRIM56 proteins, ZIKV NS5 and actin was shown in (A). Solid circle indicates a non-specific band. (B) RNA was extracted from the TRIM56-RNA immunoprecipitates, followed by quantification of ZIKV RNA levels by qPCR and normalized to input viral RNA in whole cell lysates. One-way ANOVA test, ***P<0.001. NS, not significant. Results were reproduced three times. (C) MBP-polylinker control and MBP-TRIM56-C392 proteins expressed and purified from *E*. *coli* were separated on SDS-PAGE, followed by Coomassie blue staining (left) and immunoblotting (right), respectively. Results were representative of three independent experiments. Solid circles denote degradation products of MBP-TRIM56-C392. (D and E) Two micrograms of MBP-polylinker or MBP-T56-C392 protein was incubated with 0.5 μg of ZIKV RNA at room temperature for 30 min, followed by pull-down of MBP-tagged proteins using amylose resin. After washing procedure, specifically bound proteins (D) and viral RNA (E) in pull-down complex were analyzed by immunoblotting (D) and qPCR (E), respectively. Student t-test, **P<0.01, ***P<0.001. Results were reproduced three times.

Since the NHL repeat motif of TRIM71 has been shown to bind to mRNA[28], we investigated whether TRIM56 interacts with ZIKV RNA to hamper viral RNA replication. HEK293 cells expressing control vector (Bsr), WT TRIM56, the E3-ligase-dead AA mutant, or Mut N (delta-aa 693–750) lacking a portion of the C-terminal NHL-like repeat sequence were infected by ZIKV, followed by RNP-IP of TRIM56-RNA complexes and qPCR quantifying the viral RNAs associated with TRIM56. The ectopically expressed, Flag-tagged WT and mutant TRIM56 proteins were all efficiently immunoprecipitated by the anti-Flag antibody (Fig 7A, lanes 2–4), and no background was detected in negative control (Bsr) cells (lane 1). Immunoblotting detection of ZIKV NS5 protein in input cell lysates confirmed the successful infection of ZIKV (lanes 6–9) and the antiviral effect imposed by WT TRIM56 (lane 7). Immunoblotting of the IP-enriched protein complexes showed no association of TRIM56 with viral NS5 protein or cellular actin (Fig 7A, lanes 1–4), demonstrating the specificity of the RNP-IP assay. Next, we quantified the ZIKV RNA levels co-immunoprecipitated with TRIM56 or the two TRIM56 mutants. Strikingly, significant enrichment of ZIKV RNAs was observed with WT TRIM56 or the AA mutant, but not Mut N lacking C-terminal aa 693–750 (Fig 7B, compare bars 2 and 4 vs 1, increases of ~6-8-fold). These data suggest that TRIM56 binds to viral RNA in ZIKV-infected cells via its C-terminal portion. Because the AA mutant was as efficient as WT TRIM56 in co-precipitating ZIKV RNAs (Fig 7B), we conclude that the E3 ligase activity, although indispensable for the antiviral function against ZIKV, is not required for the ability of TRIM56 to associate with viral RNAs.

To determine whether TRIM56 can directly interact with ZIKV RNA, we set up additional binding experiments in cell-free reactions using recombinant TRIM56 protein and viral RNA purified from virions. We expressed and purified from *E*. *coli* a TRIM56 fragment comprising the C-terminal 392 aa fused to MBP (MBP-T56-C392), and as a negative control, an MBP-polylinker protein. Their quality and purity were verified by Coomassie blue staining (Fig 7C, left panel) and immunoblotting (right panel) following SDS-PAGE. The recombinant proteins were incubated separately with ZIKV RNA, and thereafter the protein-RNA complexes were recovered by the MBP-tag-binding amylose resin, whereby the associated protein and RNA were isolated. Immunoblotting analysis indicated successful pull-down of the bait proteins, MBP-polylinker and MBP-T56-C392, with comparable efficiency (Fig 7D). qPCR analysis revealed that, compared with amylose resin alone or the MBP-polylinker control protein pull down groups, there was significant enrichment of ZIKV RNA by recombinant MBP-T56-C392 protein, regardless of viral RNA origin (be it MR766 or PRVABC59) (Fig 7E). These data demonstrate that TRIM56 can directly associate with ZIKV RNA via its C-terminal portion. This result is also in line with our earlier data obtained from ZIKV-infected cells (Fig 7A and 7B).

### TRIM56 restricts ZIKV in neural cells

**Fig 8:**
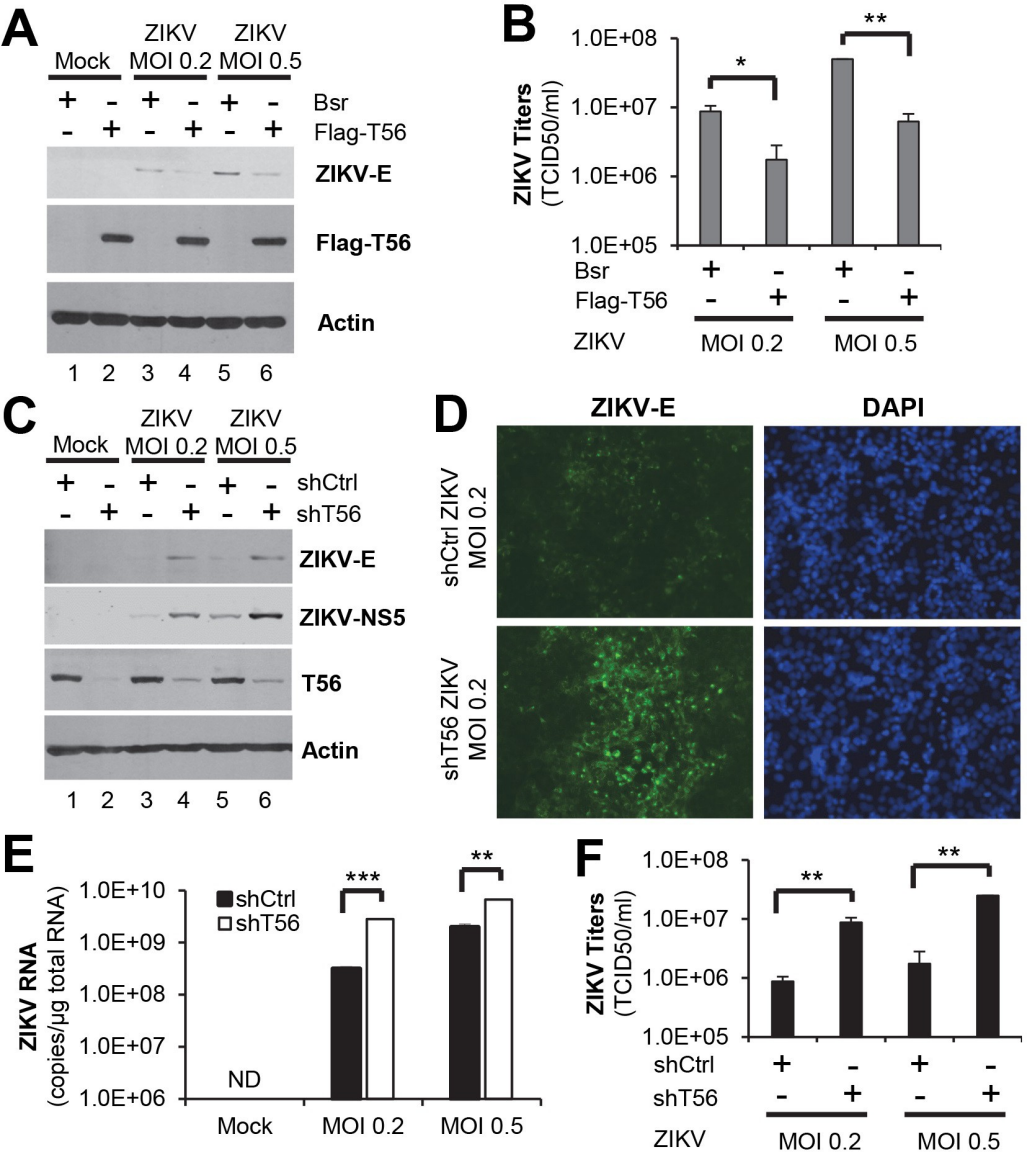
TRIM56 exhibits anti-ZIKV activity in neural cell types. (A and B) SK-N-SH cells stably transduced with empty vector (Bsr) or Flag-TRIM56 were infected by ZIKV MR766 strain at indicated MOIs for 72 h. Expression of ZIKV E, Flag-T56, and actin (loading control) proteins were determined by immunoblotting (A). Progeny virus production in culture supernatants was measured by TCID_50_ assay (B). Student t-test, *P<0.05, **P<0.01. Results were representative of three independent experiments. (C-F) SVGA cells stably transduced with non-targeting control shRNA or TRIM56 shRNA were infected with ZIKV MR766 strain at indicated MOIs for 72 h. Intracellular ZIKV E and NS5 proteins were determined by immunoblotting (C). Viral E protein expression in infected cells was also analyzed by immunofluorescence staining (D, green fluorescence). Intracellular ZIKV RNA levels (E) and extracellular progeny virus titers (F) were determined by q PCR and TCID_50_ assay, respectively. ND, non-detectable. Student t-test, **P<0.01, ***P<0.001. Results were representative of three independent experiments.

The association of ZIKV infection with fetal microcephaly highlights the importance of neurotropism in ZIKV pathogenesis. In earlier work we found two human cell lines of neural origin, SK-N-SH and SVGA, to be permissive for ZIKV (S3 Fig). These offered tractable in vitro systems for evaluating the impact of TRIM56 on ZIKV infection in neural cells. SK-N-SH cells were transduced with control vector (Bsr) or Flag-TRIM56, followed by infection by ZIKV. Immunoblotting revealed viral E protein expression was substantially reduced in cells ectopically expressing Flag-TRIM56, as compared with control Bsr cells (Fig 8A, compare lanes 4 vs 3, and 6 vs 5). In agreement with this, progeny virus titers were significantly curtailed by TRIM56 overexpression (Fig 8B, compare bars 2 vs 1, and 4 vs 3, decreases of ~6-8-fold).

To determine if endogenous TRIM56 expression restricts ZIKV in neural cells, we performed shRNA knockdown experiments in the human fetal astrocyte cell line SVGA, which expressed readily detectable TRIM56 protein. In comparison with a non-targeting control shRNA, TRIM56 shRNA significant decreased TRIM56 protein abundance (Fig 8C, compare lanes 2 vs 1). Upon infection by ZIKV, intracellular viral E and NS5 proteins accumulated to higher levels in TRIM56 knockdown SVGA cells than in cells bearing control shRNA (Fig 8C, compare lanes 4 vs 3, and 6 vs 5). Consistent with the immunoblotting data, TRIM56 depletion led to increased percentage of viral E protein-positive cells (Fig 8D), heightened intracellular viral RNA replication (Fig 8E), and elevated progeny virus production (Fig 8F). Taken together, the data gleaned from SK-N-SH and SVGA cells demonstrate that TRIM56 also functions in human cells of neural origin to impede ZIKV replication.

### ZIKV infection moderately upregulates TRIM56 expression in human neural cells and primary mouse cortical neurons

**Fig 9:**
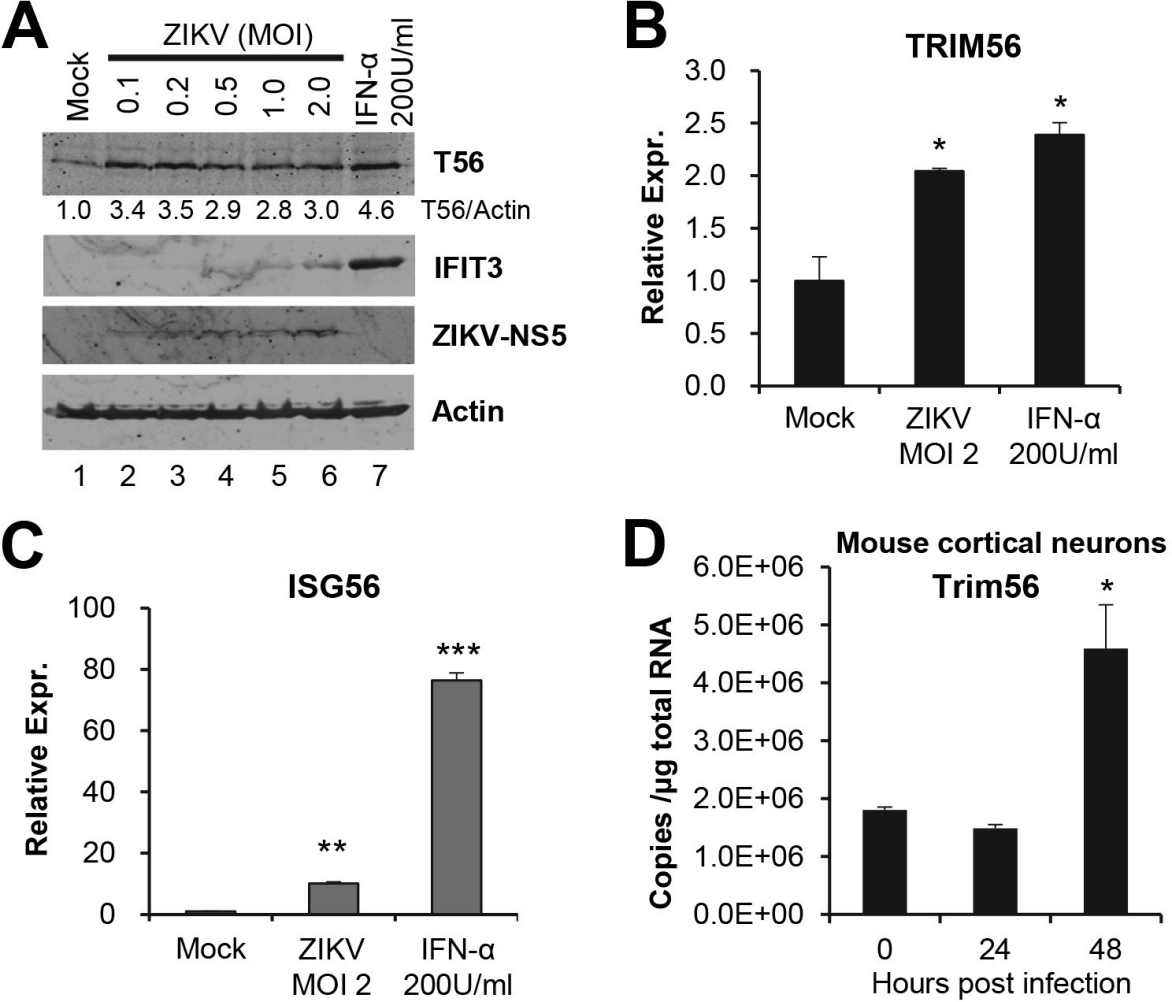
ZIKV infection moderately upregulates TRIM56 expression. (A-C) SVGA cells were infected by ZIKV MR766 strain at indicated MOIs for 72 h. IFN-α stimulation for 16 h was used as a positive control for TRIM56 induction. The expression of TRIM56, IFIT3, ZIKV NS5, and actin (loading control) proteins was determined by immunoblotting using the indicated antibodies (A). Data were representative of two independent experiments. The mRNA levels of *TRIM56* (B) and *ISG56* (C) were analyzed by qPCR. (D) qPCR analysis of *Trim56* mRNA levels in primary mouse cortical neurons infected by ZIKV PRVABC59 strain (MOI = 1) for the indicated times. Student t-test, *P<0.05, **P<0.01, ***P<0.001. Results in panels B to D were representative of two independent experiments.

To investigate if TRIM56 expression changes in neural cell types following ZIKV infection, we first examined the abundance of endogenous TRIM56 protein in SVGA cells by immunoblotting (Fig 9A). At 72 h.p.i, cells infected with increasing doses of ZIKV-MR766 all exhibited a ~3-fold increase in TRIM56 protein, compared with mock-infected cells (compare lanes 2–6 vs lane 1). This was not secondary to an overt IFN response, as minimal induction of IFIT3, a well-characterized ISG and sensitive marker of IFN production, was merely observed at high MOIs (lanes 5 and 6), and high concentration IFN treatment (lane 7) did not give rise to more robust uptick of TRIM56 expression than ZIKV, despite being a much stronger inducer of IFIT3 expression. Subsequently, qPCR of *TRIM56* and *ISG56* mRNA expression confirmed the protein data and indicated that the ZIKV upregulation of TRIM56 occurred at mRNA level (Fig 9B and 9C). To determine whether this is the case in primary cells of neural origin, we examined *Trim56* expression in primary mouse cortical neurons. Because it was challenging to obtain large number of neurons for immunoblotting, we utilized qPCR assay to quantify the mRNA levels of *Trim56* prior to and at 24 and 48 h.p.i., respectively, of ZIKV-PRVABC59 (MOI = 1). As shown in Fig 9D, these cells were found to have basal expression of *Trim56*. In line with the expression pattern of *TRIM56* transcript in SVGA cells, *Trim56* mRNA abundance was upregulated by ~ 2-fold at 48 h.p.i. In aggregate, the experiments show that TRIM56 is expressed in human and mouse neural cell types, which moderately upregulate the expression of this antiviral protein in response to ZIKV infection.

## Discussion

In this study we demonstrate that TRIM56, an E3 ubiquitin ligase ubiquitously expressed in human tissues [25], poses a barrier to ZIKV infection in human cells. Ectopic expression of TRIM56 inhibited ZIKV propagation, while depletion of endogenous TRIM56 had the opposite effect. Importantly, this was demonstrated in cells of fibroblast, epithelial and neural origins, representative cell types targeted by ZIKV in vivo. No less important, we confirmed that TRIM56 restricts both African and Asian lineages of ZIKVs. To our knowledge, this is the first study that identifies a host restriction factor of ZIKV in the TRIM protein family. Taken into account the neurotropism of ZIKV infection in vivo, our revelations that TRIM56 was basally expressed in human brain [25], human astrocytes SVGA and primary mouse neurons (this study) and that its expression was elevated following ZIKV infection in neural cell types support the biological relevance of TRIM56 as a host restriction factor of ZIKV. In agreement with our previous observation [25], TRIM56 abundance was only moderately upregulated by IFN-α or ZIKV and the increase in its expression following ZIKV infection was not strictly correlated with induction of classical ISGs (IFIT3 and ISG56), indicating different mechanism of gene expression regulation that warrants future investigation. Very recently, Seo *et al*. reported that in mouse macrophages *Trim56* deletion did not significantly alter viral RNA levels following ZIKV infection [50]. It should be noted that mouse is not a natural host of ZIKV and not susceptible to the virus unless with deficiencies in IFN induction and/or signaling [20, 21]. Consistent with this, our in vitro infection experiments demonstrated that three murine cell lines, MEFs, L929 and hepa1-6, all had limited permissiveness for ZIKV infection, as compared with human cell lines. Whether TRIM56 exhibits different roles in antiviral immunity between host species and/or between tissue origins in mouse will require additional study.

A subset of TRIM proteins has been shown to modulate innate immune signaling during viral infections [51]. Flavivirus replication generates dsRNAs, a major pathogen-associated molecular pattern that are sensed by two classes of pattern recognition receptors (PRRs), *i*.*e*., the RIG-I-like receptors (RLRs, RIG-I and MDA5) and TLR3 [52]. While TRIM56 is dispensable for RLR signaling and its overexpression per se does not trigger IFN production, it promotes TLR3-dependent innate immune response via interacting with the adaptor protein TRIF, independent of its E3 ligase activity [34]. It should be noted, however, that several lines of evidence suggest that the antiviral effect of TRIM56 against ZIKV revealed in this study is direct and not secondary to a regulation of IFN antiviral responses. First, ZIKV, like other flaviviruses, is not a strong inducer of IFN responses. Flaviviruses replicate their RNAs within re-arranged membrane structures that shield and delay viral dsRNAs from innate immune recognition [53]. ZIKV encodes multiple IFN antagonists including NS1, NS4A, NS5, among others, that inhibit IFN production and/or signaling [54–56]. Consistent with this, we found almost no induction of IFN-β, IFN-λ1 and ISGs by ZIKV in cell culture within 48 h.p.i (S7 Fig). Second, TRIM56 did not augment, but rather decreased, the minimal induction of IFNs and ISG56 late post ZIKV infection (S7 Fig), which may be explained by TRIM56-mediated reduction in ZIKV RNA (*i*.*e*., the immune stimulus) levels. Third, although ZIKV may elicit TLR3 signaling, as suggested by a recent study showing that ZIKV could disrupt neurogenesis through TLR3 activation [57], and TRIM56 promotes TLR3 signaling [34], the E3 ligase-deficient TRIM56 mutant (AA) with intact function in augmenting TLR3/TRIF signaling [34] was incapable of suppressing ZIKV propagation. Furthermore, we demonstrated that efficient knockdown of TLR3 by siRNA (S8A Fig) had no impact on the anti-ZIKV activity of TRIM56 in HEK293 cells (S8B Fig). Altogether, the anti-ZIKV action of TRIM56 is not attributed to its effect on TLR3 signaling. At present, we cannot fully exclude the possibility that in certain cell types such as immune cell subsets, TRIM56 may promote innate immune activation and fine-tune host responses, thereby adding to its direct antiviral effect. Whether this mechanism operates will require future studies.

Previously, TRIM56 has been shown to possess direct antiviral activities against distinct RNA viruses, including BVDV, YFV and DENV2 from the family *Flaviviridae*, HCoV-OC43 from the family *Coronaviridae* [24, 25], and influenza A and B viruses from the family *Orthomyxoviridae* [26]. However, the underlying mechanisms are unclear. It should be noted that TRIM56 is not a universally antiviral host factor against RNA viruses, as it had no inhibitory effects on several negative-stranded RNA viruses, including Sendai virus, vesicular stomatitis virus, and human metapneumovirus [25, 26]. In addition, TRIM56 was not found to impact replication of hepatitis C virus, a hepatotropic RNA virus classified in the family *Flaviviridae*, in permissive hepatoma Huh7 cells [25]. The present study expands the antiviral spectrum of TRIM56 to two more classical flaviviruses, ZIKV and DENV1. Mirroring the molecular determinants required for TRIM56-mediated restriction of BVDV, YFV and DENV2 [24, 25], the E3 ligase activity and the integrity of C-terminal portion were found to be both essential for the anti-ZIKV action of TRIM56. Interestingly, the very C-terminal region is also a prerequisite for TRIM56’s antiviral action against influenza viruses, although it alone was found to be sufficient in this case [26]. Notably, intracellular viral RNA replication is the molecular stage in the life cycle of all five flaviviruses at which TRIM56 targets for inhibition ([24, 25], and this study), arguing strongly a shared mechanism underlying the broad anti-flavivirus activity. As proposed previously [24, 25], TRIM56 may act on shared proviral host factor(s) for post-translational modifications such as attachment of ubiquitin or ubiquitin-like modifier linkages via its E3 ligase activity, in ways that alter their turn-over rates, trafficking to and/or incorporating into viral replicase complexes. The possibility that TRIM56 binds to viral RNAs via its C-terminal region to hinder viral RNA replication was also raised [25]. As discussed below, we favor the hypothesis that these two scenarios are not mutually exclusive and they operate concurrently and/or in concert to implement viral restriction.

Importantly, the current study significantly advances our understanding of the antiviral mechanism of TRIM56 by showing that TRIM56 is an RNA-binding protein and that its C-terminal region mediates the association with ZIKV RNA in infected cells. In addition, we revealed that host miRNAs are not essential for ZIKV replication and that the restriction of ZIKV by TRIM56 is independent of miRNA biogenesis or its regulation. TRIM56 is currently classified within the subgroup C-V of TRIM proteins [58], because its C-terminal region was not found to contain a defined protein domain structure. However, we previously found aa 521–748 in the C-terminal portion of TRIM56 constitutes a NHL-like domain that shares homology and conserved residues with the NHL repeat domain of several TRIM-NHL proteins such as TRIM2, TRIM3, and TRIM71 [26]. The NHL repeat domain folds into a six-bladed beta-propeller resembling that of WD40 domains [59]. Part of its surface is positively charged and may potentially interact with nucleic acids, whose backbone is negatively charged. In line with this model, TRIM71 associates with miRNA and Argonaute2 via its NHL domain, thereby regulating miRNA function and gene expression [60]. The NHL domain also targets TRIM71 to cellular mRNAs, leading to repression of gene expression [28]. Additionally, NHL repeats confer binding affinity of other TRIM-NHL proteins to cellular miRNAs and mRNAs [27–29]. Altogether, these prompted us to determine if miRNA regulation and/or RNA-binding play a part in the anti-ZIKV action of TRIM56. To assess the potential connection of TRIM56 with miRNA, we investigated the impact of TRIM56 on ZIKV infection in Dicer knockout cells that lack the biogenesis of mature miRNAs. We did not observe any influence of host miRNA deficiency on TRIM56-imposed suppression of ZIKV RNA replication, viral protein expression, or progeny virus production, suggesting a miRNA-independent antiviral mechanism. We subsequently tested if TRIM56 could bind to viral RNA in infected cells. Indeed, a specific association between TRIM56 and ZIKV RNA was detected in RNP-IP analysis. In contrast, a TRIM56 mutant lacking aa 693–750, the very C-terminal end portion of the NHL-like domain, was no longer able to capture viral RNA, concomitant with a loss in antiviral activity. Importantly, we have demonstrated that a recombinant TRIM56 fragment composed of the C-terminal 392 aa was able to efficiently capture ZIKV RNA in cell-free reactions, suggesting the TRIM56-viral RNA interaction is direct and does not depend on cellular factors, although we cannot exclude the possibility that certain cellular proteins may fine-tune this interaction in infected cells. Interestingly, the E3 ligase activity, another key anti-ZIKV determinant, was found to be dispensable for TRIM56-ZIKV RNA association. Taken together, these observations imply that the full anti-ZIKV action of TRIM56 necessitates not only the C-terminal RNA-binding region to recognize ZIKV RNA, but also the activity of E3 ligase that catalyzes post-translational modification(s) of pro-viral host (or viral) factors to impede their involvement/function in viral RNA replication (S9 Fig). Given previous reports that TRIM5, TRIM25, and TRIM6, can synthesize unanchored polyubiquitin chains via their E3 ligase activities [61–63] and that unanchored polyubiquitin chains play a role in the life cycle of an RNA virus (influenza A virus) [64], there is a possibility that TRIM56 may catalyze the synthesis of unanchored polyubiquitin chains to regulate the flaviviral RNA replication process. We also cannot rule out the possibility that TRIM56 promotes ubiquitin-like modifications, such as sumoylation and ISGylation that were reported with TRIM28 and TRIM25 [65, 66], respectively, to execute its antiviral function.

While future studies are needed to define the boundary of the RNA-binding motif(s) within TRIM56, we favor a model in which the structured NHL-like domain mediates the interaction with viral RNA. Lending support to this notion, our previous studies have shown that the integrity of the TRIM56 C-terminal portion is essential for inhibiting viral RNA replication of BVDV, DENV2 and YFV, and that two different C-terminal deletion mutants, Δaa 621–695 and Δaa 693–750, each lacking a separate portion of the NHL-like domain, invariably lost antiviral activity [24, 25]. Whether TRIM56 binds to these flaviviral RNAs via its C-terminal NHL-like domain to exert the observed antiviral effects, as it does with ZIKV RNA, warrants further investigation.

In summary, the present study identifies TRIM56 as a novel restriction factor of ZIKV, deepening our understanding of host intrinsic mechanisms that fend off this medically important flavivirus and broadening the antiviral spectrum of TRIM56. Moreover, the revelation that TRIM56 is an RNA-binding protein that associates with viral RNAs in ZIKV-infected cells and in cell-free reactions unravels new insights into the molecular mechanisms by which TRIM56 restricts flaviviruses. Further investigations that elucidate the precise action mechanism of TRIM56 may expose therapeutic targets that can be harnessed to combat ZIKV and possibly other flavivirus infections.

## Supporting information

S1 Fig ZIKV susceptibility of cell lines of human hepatocyte origin. ZIKV E protein was immunostained with green fluorescence, and nuclei were counterstained blue with DAPI. Images were representative of two independent experiments.
(TIF)

S2 Fig HeLa-FitA2 cells support ZIKV infection. ZIKV E protein was immunostained with green fluorescence, and nuclei were counterstained blue with DAPI. Images were representative of three independent experiments.
(TIF)

S3 Fig Human neural cell lines (SK-N-SH and SVGA) are susceptible to ZIKV infection. ZIKV E protein was immunostained with green fluorescence, and nuclei were counterstained blue with DAPI. Images were representative of two independent experiments.
(TIF)

S4 Fig Permissiveness of non-human cell lines (BSC-1 and MDCK) for ZIKV. ZIKV E protein was immunostained with green fluorescence, and nuclei were counterstained blue with DAPI. Images were representative of two independent experiments.
(TIF)

S5 Fig Differential susceptibility to ZIKV infection of murine and human cell lines. The indicated cell lines were infected by ZIKV MR766 strain (MOI = 1) for 24 h or 48 h, followed by qPCR analysis of intracellular viral RNA levels. Data were representative of two independent experiments.
(TIF)

S6 Fig TRIM56 inhibits DENV-1 RNA replication. Replication of a luciferase-encoding DENV-1 RNA replicon in HEK293-FIT-T56 cells repressed (Dox-) or induced (Dox+) for HA-TRIM56 expression at different times post electroporation. Student t-test, **P<0.01. Results were representative of three independent experiments.
(TIF)

S7 Fig Ectopic expression of TRIM56 does not enhance ZIKV-induced innate immune response. HEK293-T3Y cells with and without expression of Flag-HA-TRIM56 (FH-T56) were infected by ZIKV for the indicated times, followed by qPCR analysis of the expression of *RANTES* (A), *ISG56* (B), *IFNB* (C) and *IL29* (D). Results were representative of three independent experiments.
(TIF)

S8 Fig Knockdown of TLR3 does not affect the anti-ZIKV activity of TRIM56. HEK293 cells expressing control vector (Bsr) or Flag-T56 were transfected with non-targeting control siRNA or TLR3 siRNA for 24 h, followed by infection by ZIKV-MR766 for additional 48 h. The expression of *TLR3* mRNA (A) and intracellular viral RNA levels (B) were quantified by qPCR. Student t-test, **P<0.01, ***P<0.001. Results were representative of two independent experiments.
(TIF)

S9 Fig Graphic abstract of the findings of this study. TRIM56 binds to ZIKV RNA via its C-terminal portion, in ways that involve its E3 ligase activity to impede viral RNA replication.
(TIF)
