# Supplementary figures and images for: The E3 ligase TRIM56 is a host restriction factor of Zika virus and depends on its RNA-binding activity but not miRNA regulation, for antiviral function
Source: PLoS Negl Trop Dis. 2019 Jun 28;13(6):e0007537. doi: 10.1371/journal.pntd.0007537 (PMC6623546; doi:10.1371/journal.pntd.0007537)

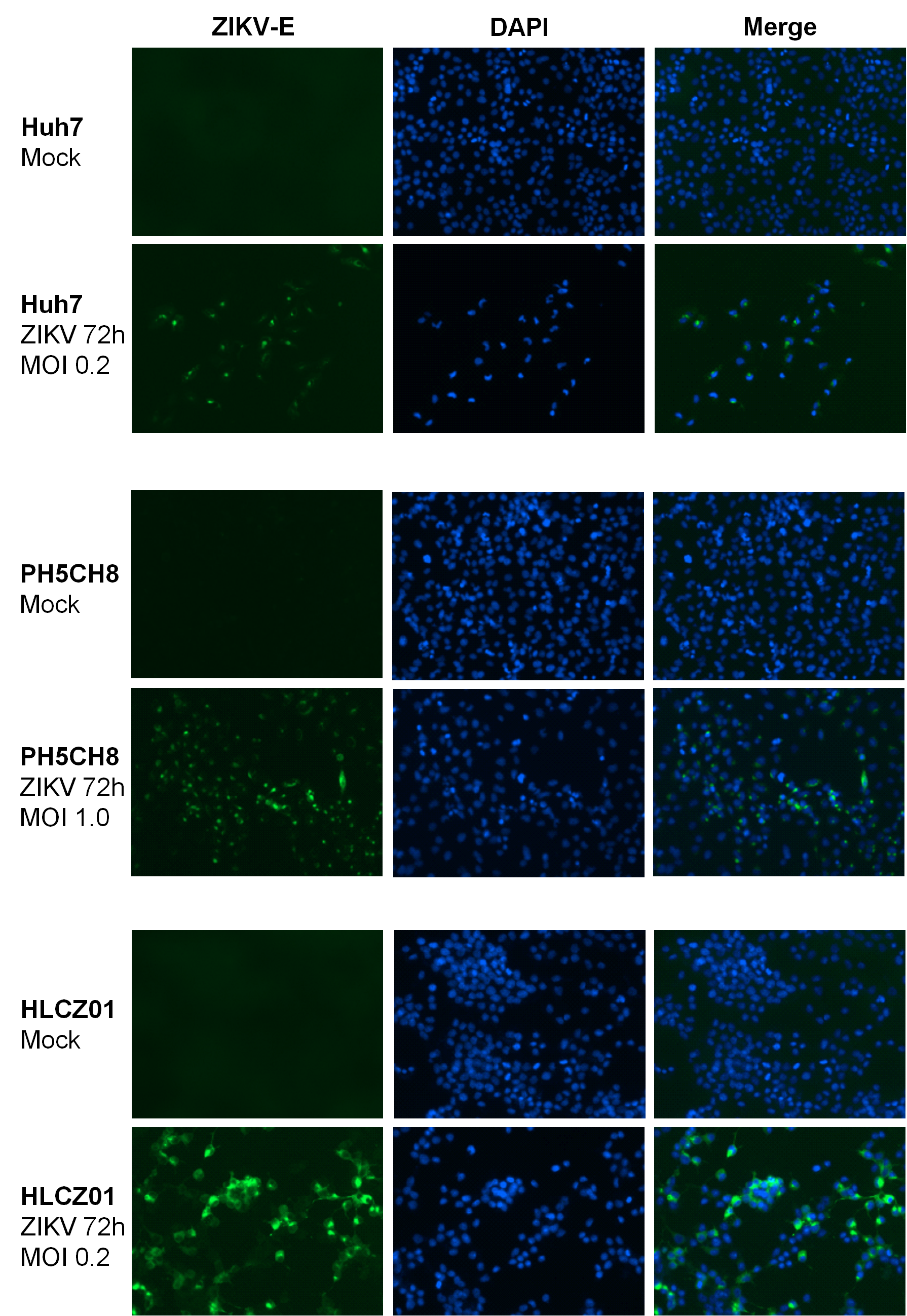

Supplement: S1 Fig — ZIKV E protein was immunostained with green fluorescence, and nuclei were counterstained blue with DAPI. Images were representative of two independent experiments. (TIF) [file pntd.0007537.s001.tif]

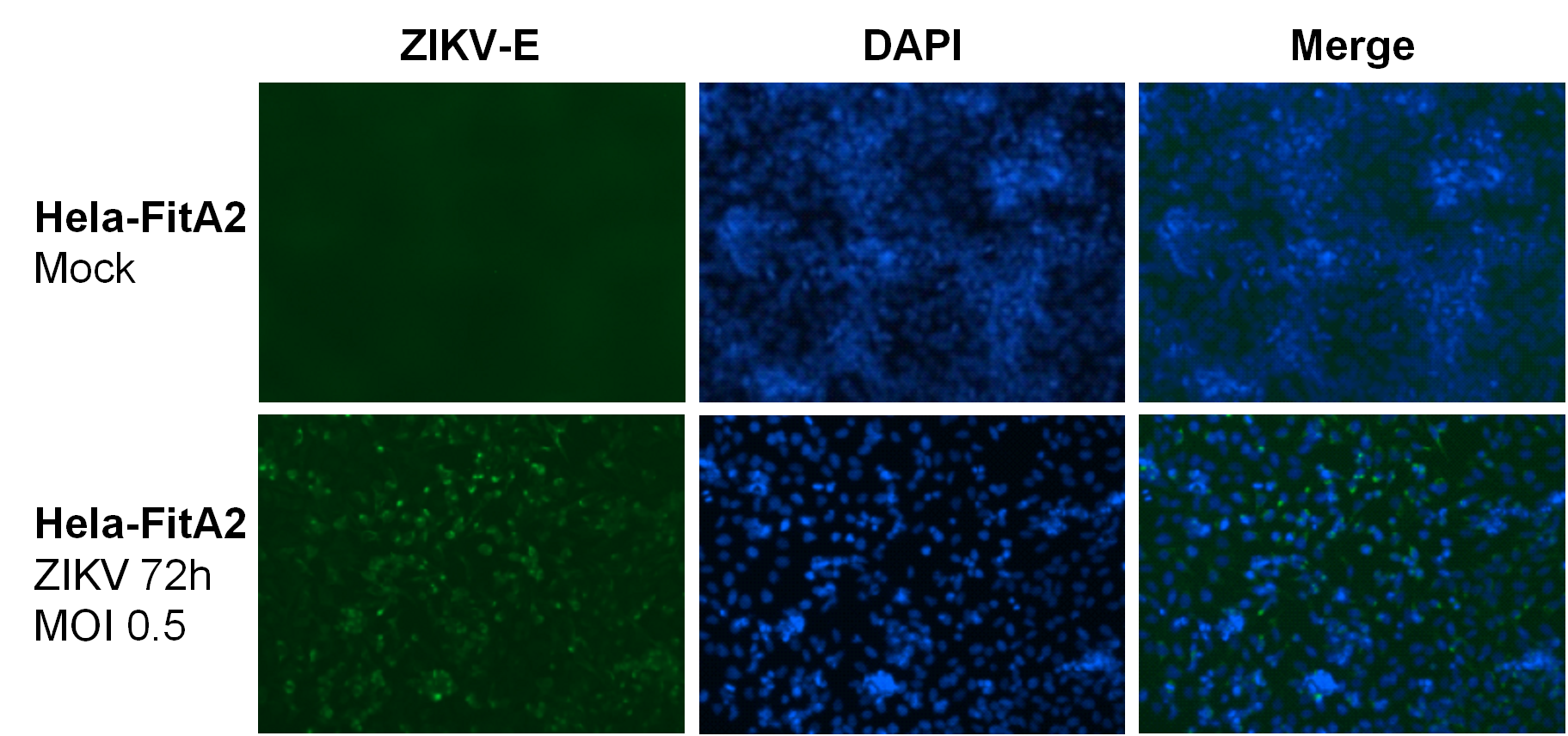

Supplement: S2 Fig — ZIKV E protein was immunostained with green fluorescence, and nuclei were counterstained blue with DAPI. Images were representative of three independent experiments. (TIF) [file pntd.0007537.s002.tif]

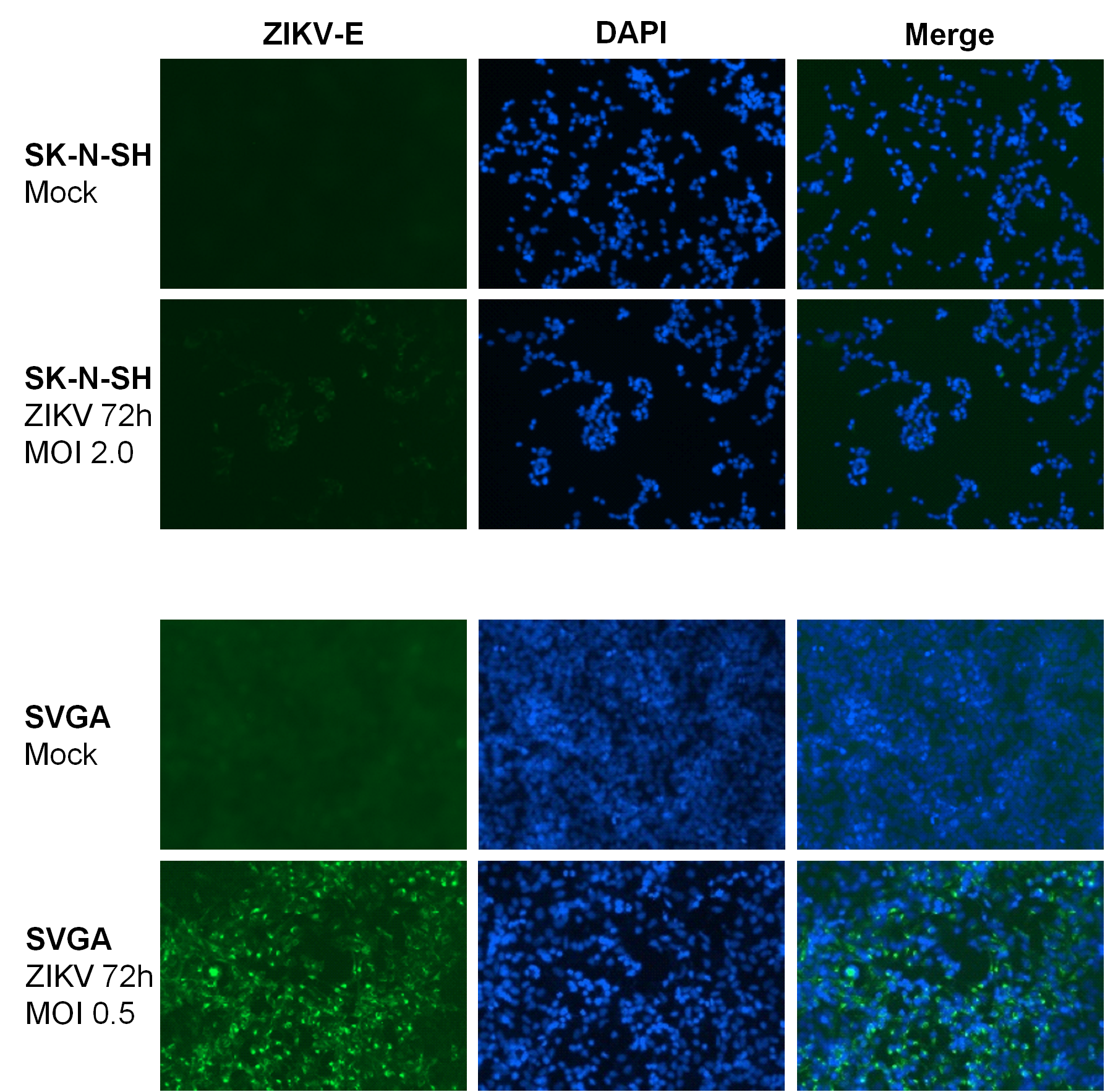

Supplement: S3 Fig — ZIKV E protein was immunostained with green fluorescence, and nuclei were counterstained blue with DAPI. Images were representative of two independent experiments. (TIF) [file pntd.0007537.s003.tif]

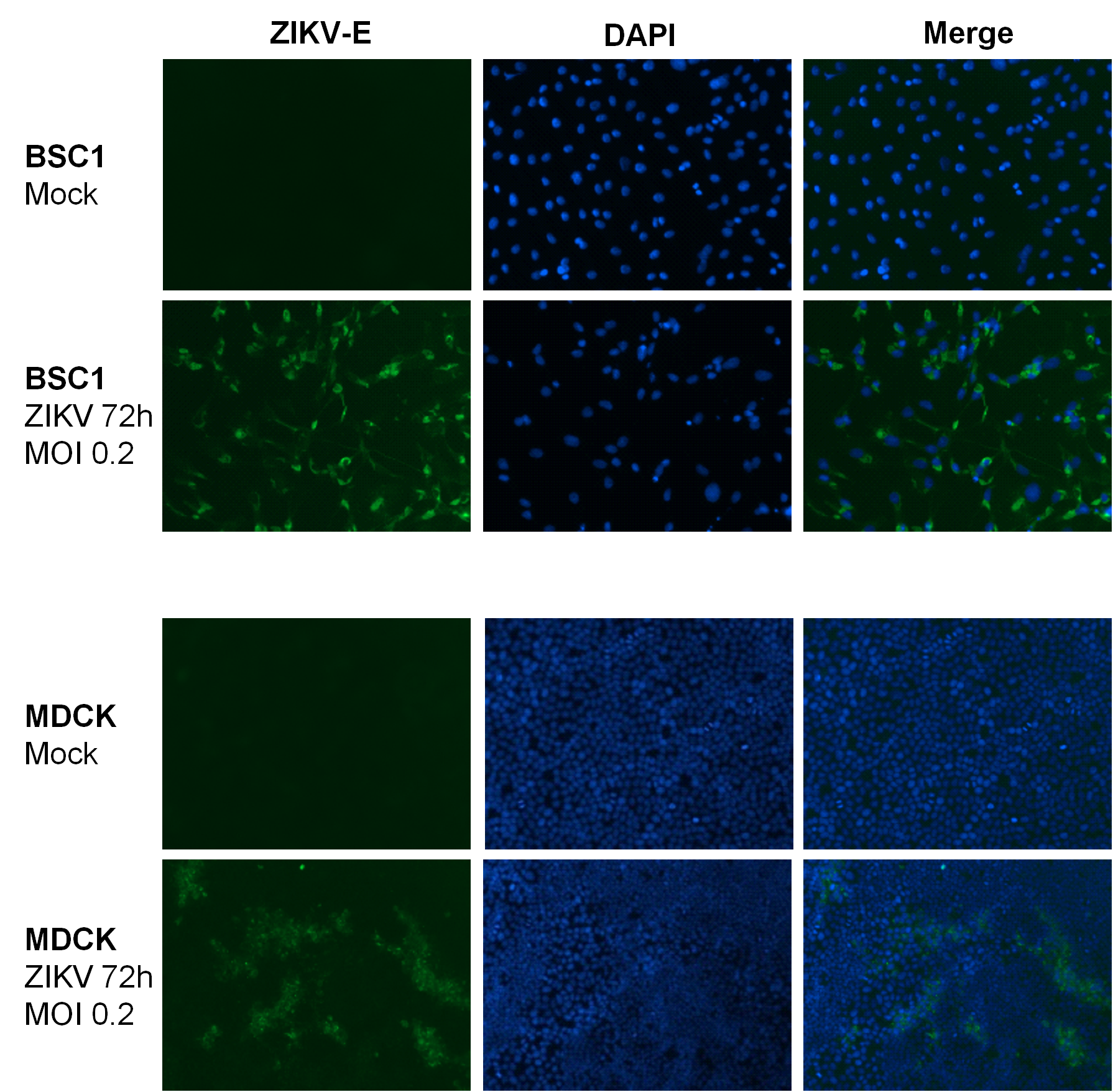

Supplement: S4 Fig — ZIKV E protein was immunostained with green fluorescence, and nuclei were counterstained blue with DAPI. Images were representative of two independent experiments. (TIF) [file pntd.0007537.s004.tif]

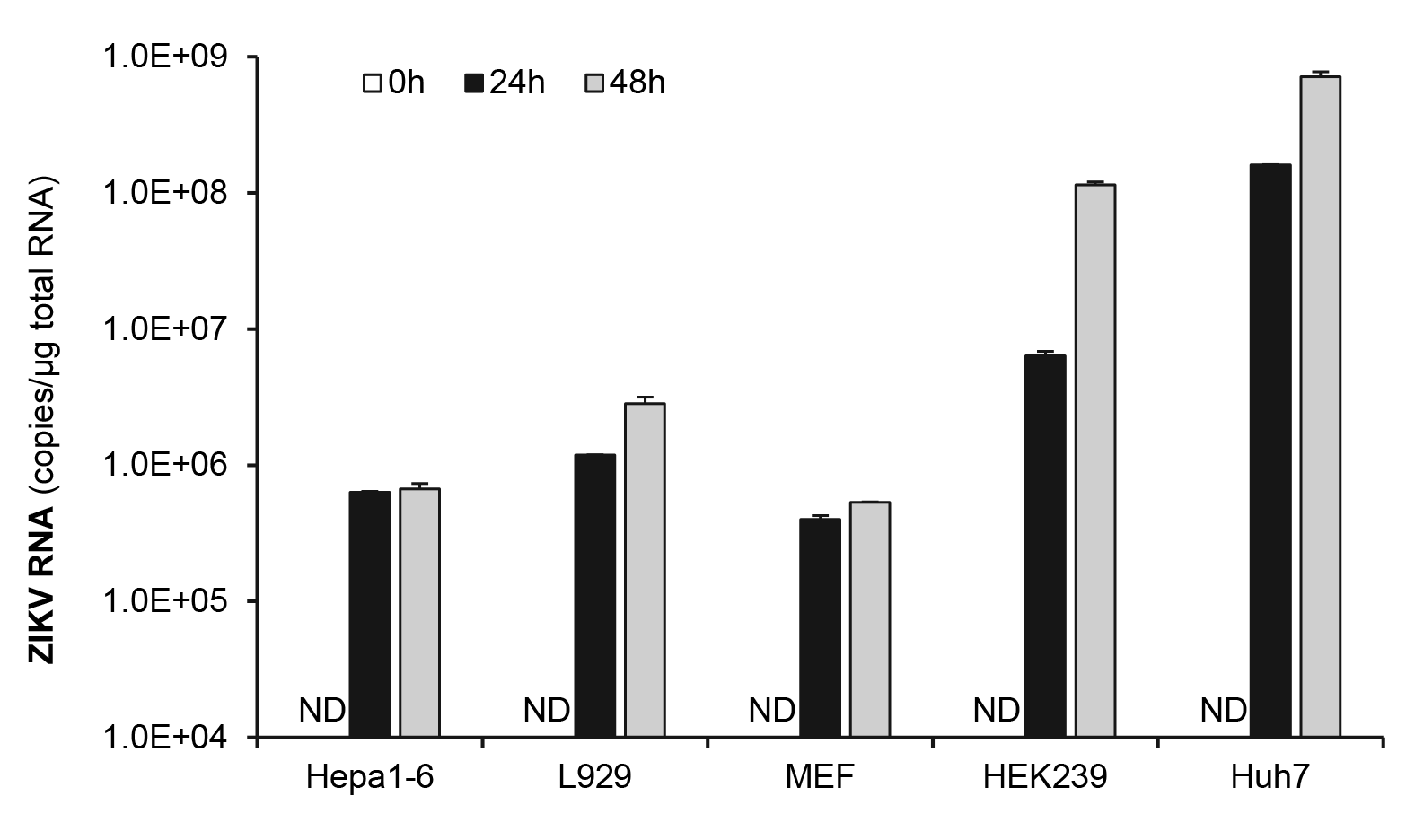

Supplement: S5 Fig — The indicated cell lines were infected by ZIKV MR766 strain (MOI = 1) for 24 h or 48 h, followed by qPCR analysis of intracellular viral RNA levels. Data were representative of two independent experiments. (TIF) [file pntd.0007537.s005.tif]

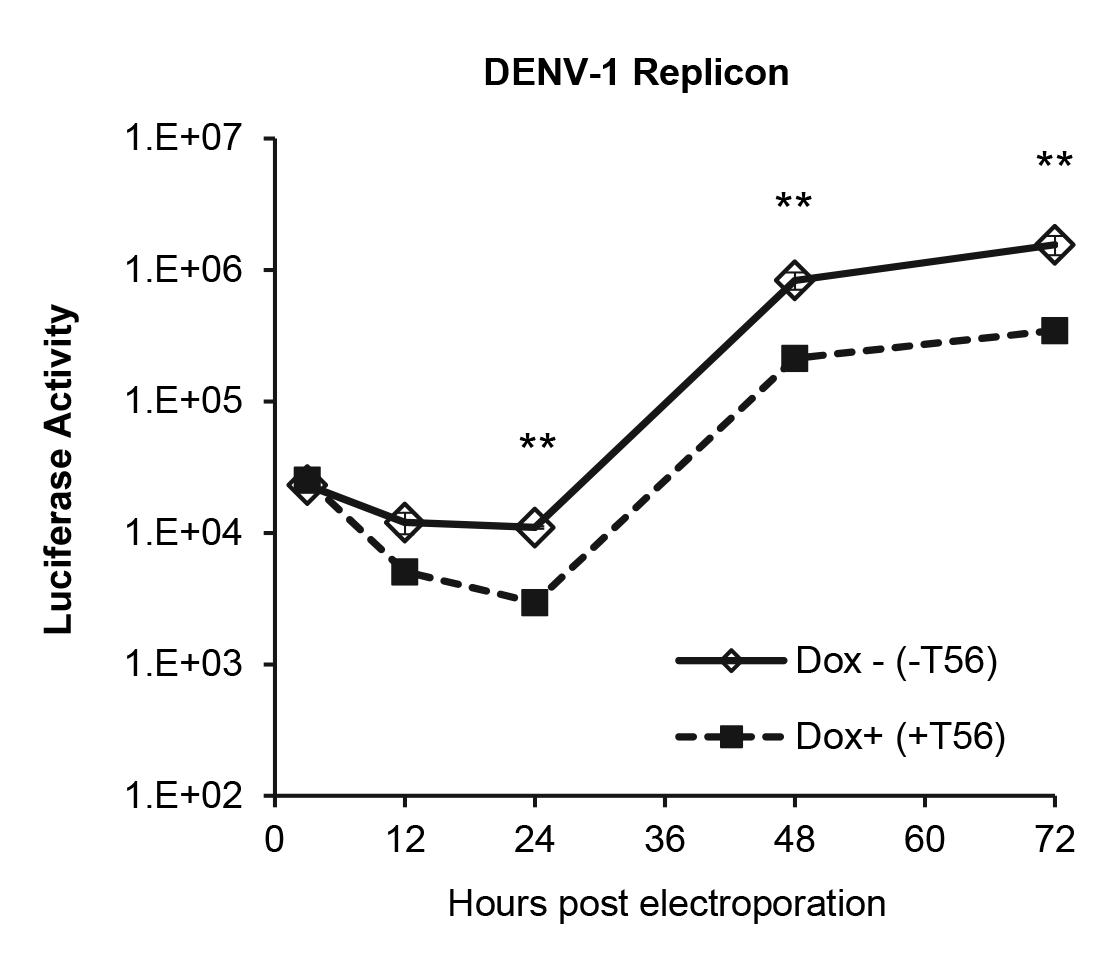

Supplement: S6 Fig — Replication of a luciferase-encoding DENV-1 RNA replicon in HEK293-FIT-T56 cells repressed (Dox-) or induced (Dox+) for HA-TRIM56 expression at different times post electroporation. Student t-test, **P<0.01. Results were representative of three independent experiments. (TIF) [file pntd.0007537.s006.tif]

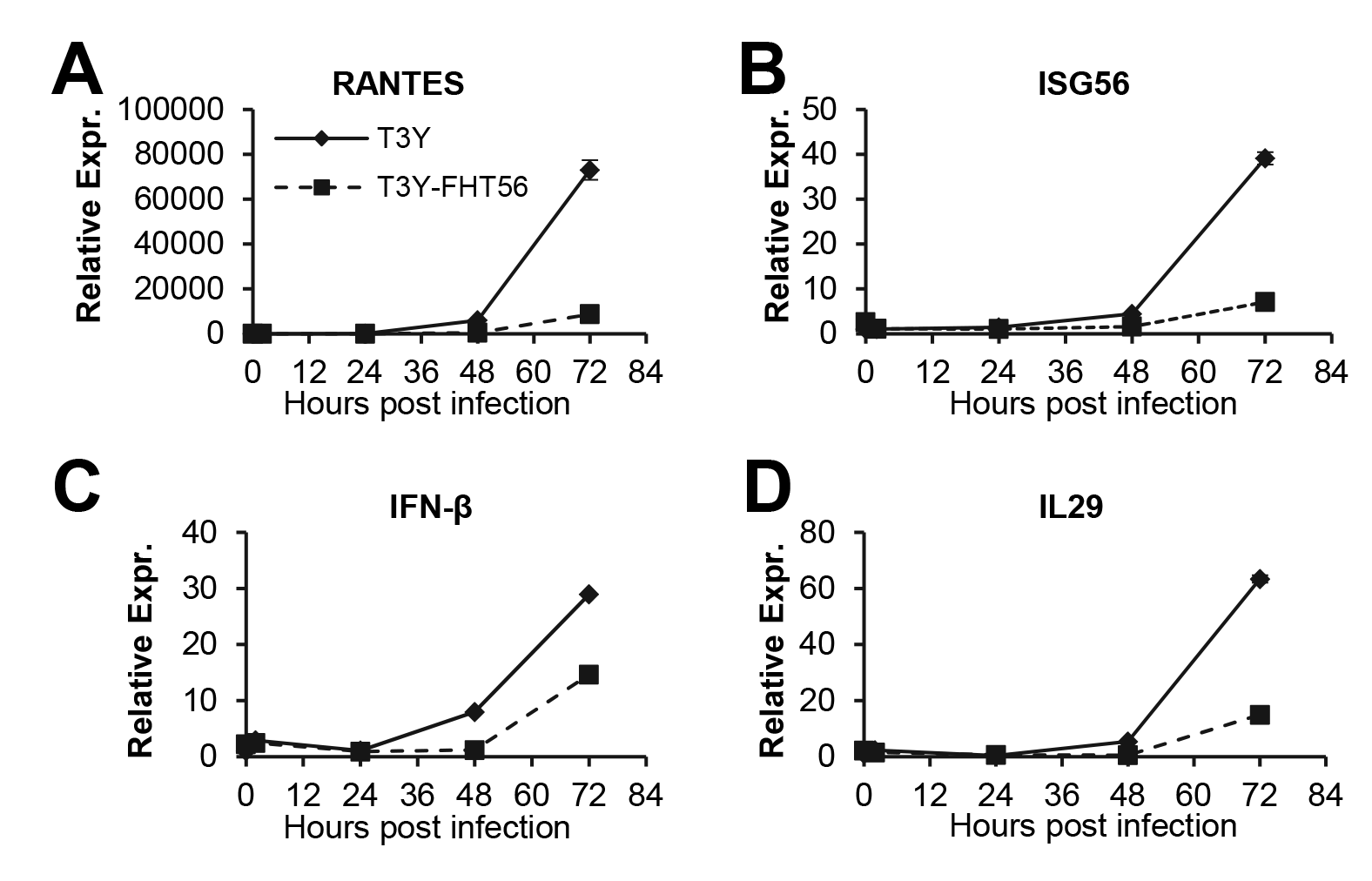

Supplement: S7 Fig — HEK293-T3Y cells with and without expression of Flag-HA-TRIM56 (FH-T56) were infected by ZIKV for the indicated times, followed by qPCR analysis of the expression of RANTES (A), ISG56 (B), IFNB (C) and IL29 (D). Results were representative of three independent experiments. (TIF) [file pntd.0007537.s007.tif]

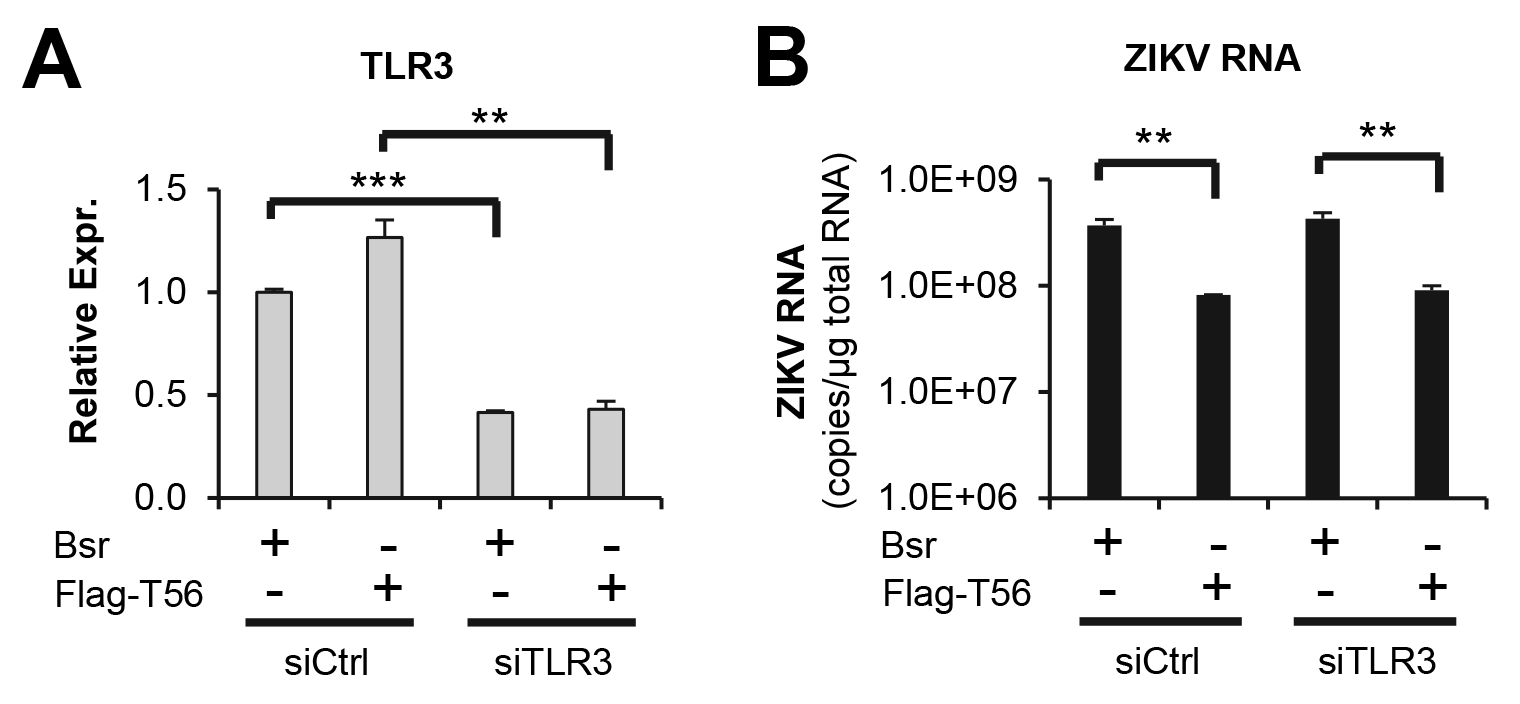

Supplement: S8 Fig — HEK293 cells expressing control vector (Bsr) or Flag-T56 were transfected with non-targeting control siRNA or TLR3 siRNA for 24 h, followed by infection by ZIKV-MR766 for additional 48 h. The expression of TLR3 mRNA (A) and intracellular viral RNA levels (B) were quantified by qPCR. Student t-test, **P<0.01, ***P<0.001. Results were representative of two independent experiments. (TIF) [file pntd.0007537.s008.tif]

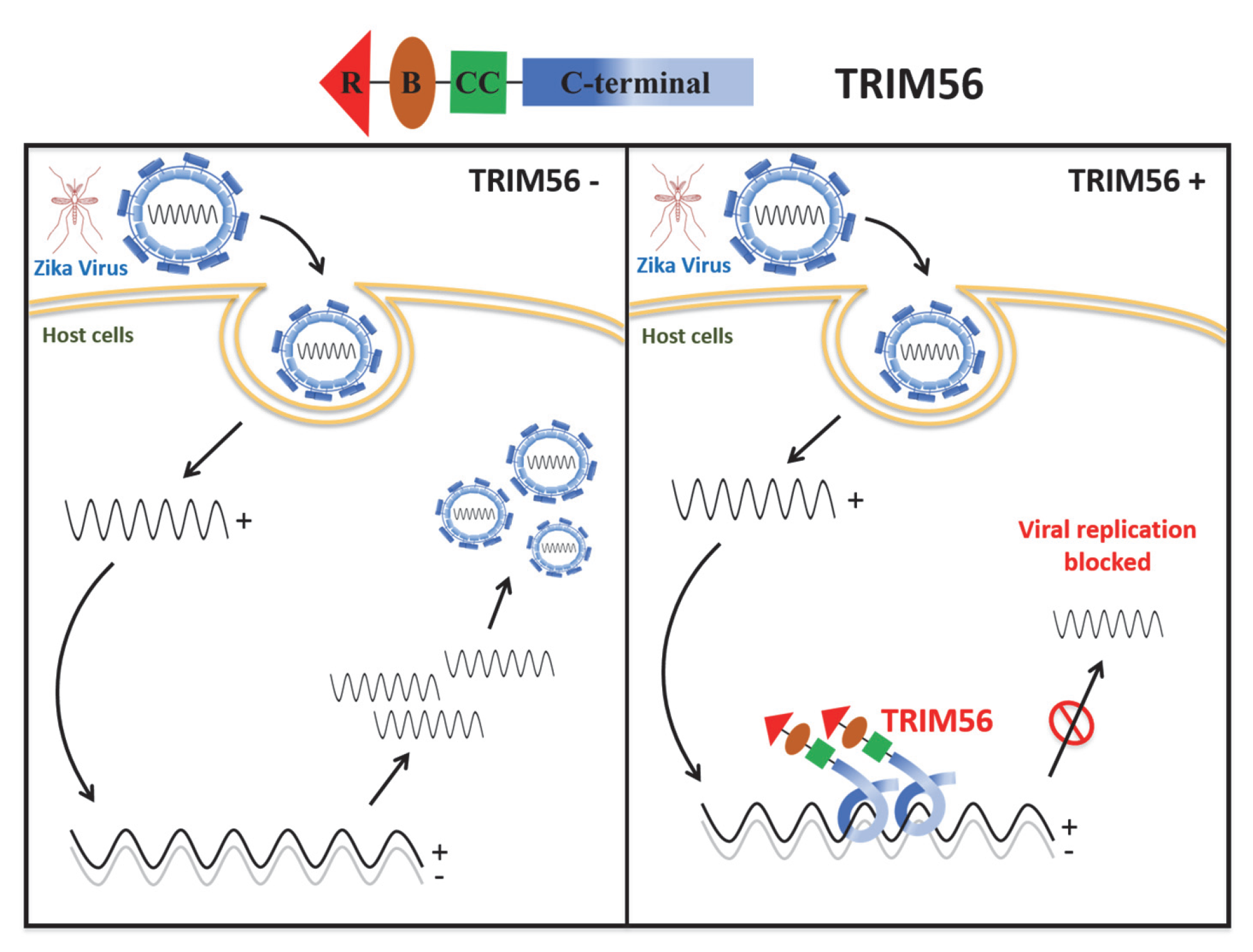

Supplement: S9 Fig — TRIM56 binds to ZIKV RNA via its C-terminal portion, in ways that involve its E3 ligase activity to impede viral RNA replication. (TIF) [file pntd.0007537.s009.tif]
